# Japanese written pseudowords can be conditioned to Japanese spoken words with positive, negative, and active emotions

**DOI:** 10.1007/s10339-023-01138-0

**Published:** 2023-07-14

**Authors:** Misa Ando, Toshimune Kambara

**Affiliations:** Department of Psychology, Graduate School of Humanities and Social Sciences, Hiroshima University, Higashihiroshima, Japan

**Keywords:** Linguistic conditioning, Evaluative conditioning, Classical conditioning, Association, Japanese language

## Abstract

This study aimed to examine whether Japanese participants condition spoken words’ meanings to written pseudowords. In Survey 1, we selected spoken words associated with negative (*α* = .91) and positive (*α* = .79) features for Experiment 1 and passive (*α* = .90) and active (*α* = .80) features for Experiment 2. In Experiment 1, participants evaluated four written pseudowords’ emotional valence using a 7-point semantic differential scale (1: *negative*; 7: *positive*) before and after conditioning spoken words with negative, neutral, or positive features to each pseudoword. In the conditioning phase, participants read each pseudoword, listened to a spoken word, and verbally repeated each spoken word. The results showed that a pseudoword was conditioned to spoken words with positive and negative features. In Experiment 2, participants evaluated four pseudowords’ activeness using a 7-point semantic differential scale (1: *passive*; 7: *active*) before and after conditioning spoken words of passive, neutral, and active features to each written pseudoword. In the conditioning phase, the participants read each written pseudoword, listened to a spoken word, and repeated the spoken word. The results showed that the activeness evaluations were more increased for pseudowords conditioned to spoken words of active and neutral features after conditioning than before conditioning but were unchanged for a pseudoword conditioned to those with passive features before and after conditioning. Additonally, Survey 2’s results showed that although the positiveness and activeness responses of the words used in Experiments 1 and 2 were controlled well, the lack of significant differences among positiveness responses of words may influence the evaluative conditioning in Experiment 2. That is, when participants condition passive (low arousal) words’ activeness (arousal) ratings to those of pseudowords, words’ positiveness (valence) ratings would be important in the evaluative conditioning. Our findings suggest that participants can condition spoken word meanings of preference and activeness to those of written pseudowords. It also indicates that linguistically evaluative conditioning’s effects are robust in a non-alphabetic language.

## Introduction

Language is a principle characteristic of humanity (Staats 2012). As a minimally meaningful unit in language, a word comprises an association between perceptually linguistic (i.e., written and spoken word forms, Braille, fingerspelling, and so on) and perceptually and emotionally referential information (Kambara et al. 2020; Paivio 1986). The association between linguistic and referential information can be arbitrary (e.g., de Saussure 1959) and nonarbitrary (e.g., Blasi et al., 2016). An evidence-based theory suggests that the linguistic and referential features were processed in two differential systems, including verbal and nonverbal systems (e.g., Paivio 1986). Many previous studies have reported that the association between linguistic and referential information can be learned through our perceptual and emotional experiences in the first and second languages (Breitenstein et al. 2005; Carpenter and Olson 2012; Cornelissen et al. 2004; Grönholm et al. 2005; Havas et al. 2018; Hawkins et al. 2015; Hawkins and Rastle 2016; Horinouchi et al. under revision; Hultén et al. 2009; Jeong et al. 2010; Kambara et al. 2013; Lee et al. 2003; Li et al. 2020; Liu et al. 2021; Takashima et al. 2017; Tsukiura et al. 2010, 2011; Yan et al. 2021; Yang et al. 2021). Other studies have found that participants could condition subjective evaluations (unconditioned responses: UCR) of real words and perceptual stimuli (unconditioned stimuli: UCS or US) to those (conditioned responses: CR) of other real words, pseudowords, symbols, and other perceptual stimuli (conditioned stimuli: CS, e.g., Barnes-Holmes et al. 2000; Cicero and Tryon 1989; Hughes et al. 2018; Paivio 1964; Staats and Staats 1958a; Staats et al. 1961; Staats and Staats 1957; Till and Priluck 2000, 2001; Tryon and Cicero 1989; Valdivia-Salas et al. 2013; for review, De Houwer et al. 2001; Jaanus et al. 1990). The phenomenon, in which people condition evaluations of stimuli to those of other stimuli, has been termed evaluative conditioning (Hofman et al. 2010). Evaluative conditioning can be conducted in three emotionality dimensions, including evaluation (valence: ratings from *pleasant* to *unpleasant*), activity (arousal: ratings from *active* to *passive*), and potency (dominance: ratings from *strong* to *weak*). These dimensions are associated with semantic differential methods (Osgood et al. 1957) and Self-Assessment Manikin (Bladley and Lang 1984). The verbally evaluative conditioning (learning) can affect the evaluative meanings (evaluative responses) of words (Jaanus et al. 1990). As pioneers of the verbally evaluative conditioning, Staats and Staats (1957) showed that native English speakers conditioned subjective evaluations (meanings) of spoken English words to those of English pseudowords in the three dimensions (evaluation, potency, and activity). Basically, the pseudowords as linguistic information would not be associated with any sensorimotor or emotional features as nonverbal information in the mental lexicon (e.g., Fritsch and Kuchinke 2013). However, the pseudowords may include sound symbolic effects (e.g., Davis et al. 2019; Lupyan and Casasanto 2015; Spence and Gallace 2011). Additionally, previous research shows that participants conditioned subjective evaluations of real English words to those of other real English words (e.g., Staats and Staats 1958a; Staats and Staats 1958b). These results suggest that the evaluative conditioning can reorganize the associations between the linguistic and referential information in the mental lexicon. The reorganization of the mental lexicon indicates that the affective meanings of words would be reconfigured by the evaluative conditioning. Evaluative conditioning affects the lexical access of words (Kuchinke and Mueller 2019) because lexical access is faster to affective than neutral words (Kissler and Herbert 2013). The effects of evaluative conditioning were higher for the supraliminal (conscious) than for the subliminal (unconscious) presentation of UCS (US) and for self-report than for implicit methods (Hofmann et al. 2010). A study of a non-alphabetic language showed that people can unconsciously (subliminally) condition words to other words (Galli and Gorn 2011). However, many studies of alphabetic languages show the same (e.g., De Houwer et al. 1994, 1997; Dijksterhuis 2004). A recent study of Brazilian samples has also reported that although the subliminally and verbally evaluative conditioning did not affect CS evaluation, people exposed to eating-related words (CS) and positive word pairings had increased saliva compared with those conditioned to CS and negative word pairings (Passalli et al. 2022). Most of the previous findings suggest that native speakers of alphabetic languages could associate subjective evaluations (meanings) of UCS, including alphabetically real words, to those (meanings) of CS, including alphabetic pseudowords. However, to the best of our knowledge, no study examines whether native speakers of non-alphabetic languages can consciously condition words to other words, including pseudowords, as shown in Staats and colleagues’ studies in English (Staats and Staats 1957; Staats et al. 1959). A recent study has shown that although the verbally and consciously (supraliminally) evaluative conditioning occurred in an alphabetic native language (Spanish) and non-alphabetic second languages (Chinese and Japanese), the effect of evaluative conditioning was higher for the native language than for the second languages (Vidal et al. 2021). Because native speakers of alphabetic languages can consciously condition evaluative responses of words to other words in both the alphabetically native and non-alphabetical second languages, the native speakers of a non-alphabetical language would also condition evaluative responses (meanings) of words as UCS (e.g., positive/negative, active/passive, or strong/weak meanings of words; Staats and Staats 1957) to those of pseudowords as CS by consciously evaluative conditioning in non-alphabetic languages. Regarding evaluative conditioning of language, one of the important things would be linked to whether or not participants have evaluative responses to words used as UCS. Additionally, the features of evaluative responses of words used as UCS (i.e., evaluation, potency, and activity) would be essential for the evaluative conditioning of language. However, differences among first (native) languages would not influence the evaluative conditioning of language for native speakers. Nonetheless, differences between first and second languages would affect the evaluative conditioning of language (Vidal et al. 2021). This study aims to clarify whether native speakers of a non-alphabetic language (Japanese) condition the subjective evaluations of spoken Japanese words to those of written Japanese pseudowords. Japanese language is among the many non-alphabetic languages. Before this study, we predicted that native Japanese speakers condition the meanings of spoken Japanese words as UCS to those of written Japanese pseudowords used as CS based on previous research of evaluative conditioning of language (language conditioning, e.g., Staats 2012). The Japanese writing system includes katakana (カタカナ), hiragana (ひらがな), and kanji characters (漢字), with roman letters (ローマ字), numbers, and symbols also being used (Yamaguchi 2007). In Experiments 1 and 2, we used katakana, which is among the original and non-alphabetic characters in Japanese, as written CS.

## Methods

We examined whether subjective evaluations of written Japanese pseudowords were conditioned to those of spoken Japanese words. First, we conducted a survey study (Survey 1) in which participants evaluated whether each presented word was positive or negative, active or passive, and strong or weak. The Japanese words were translated from the English words in Staats and Staats’s study (1957). In Survey 1, we used five-point scales for preliminary checking of the word evaluations. Second, after selecting the word stimuli, we conducted two behavioral experiments (Experiments 1 and 2). In Experiment 1, we examined whether subjective evaluations of positive and negative words were conditioned to those of pseudowords. In Experiment 2, we examined whether subjective evaluations of active and passive words were conditioned to those of pseudowords. Lastly, a post-hoc survey (Survey 2) was conducted to specify evaluations (both the positive/negative and active/passive ratings) of all the words used in Experiments 1 and 2 because Survey 1 only specified positive/negative ratings of positive and negative words for Experiment 1 and active/passive ratings of active and passive words for Experiment 2. Survey 2 clarified all the ratings of the words used in Experiments 1 and 2. The surveys (Surveys 1 and 2) and behavioral experiments (Experiments 1 and 2) according to the Declaration of Helsinki were approved by the ethical committee of the Graduate School of Humanities and Social Sciences at Hiroshima University. Additionally, we obtained informed consents from participants who pressed a key to approve each participation after reading an explanation of the surveys (Surveys 1 and 2) and behavioral experiments (Experiments 1 and 2).

### Survey 1

We conducted Study 1 to select Japanese real words translated from English real words used by Staats and Staats (1957) and Staats et al. (1959). Based on Cronbach’s alphas in each category of real words, we selected Japanese real words associated with negative and positive features for Experiment 1 and those associated with passive and active features for Experiment 2. However, we decided not to use Japanese real words associated with weak and strong features for a behavioral experiment since the Cronbach’s alphas of these words did not show sufficient reliability (Cronbach’s alphas) higher than 0.70 (Cortina 1993).

### Participants

Eighty-four undergraduate students, who attended a psychological lecture, participated in this survey study (26 females; *M*_*age*_ = 19.21; *SD*_*age*_ = 1.37). All the participants were healthy native Japanese speakers.

### Stimuli

We translated 97 Japanese real words used by Staats and Staats (1957) and Staats et al. (1959) to Japanese real words using a dictionary (Minamide 2014). The previous studies (Staats and Staats 1957; Staats et al. 1959) collected most of the relevant words from Osgood and Suci (1955), who showed that the bipolar word pairs (e.g., old and young as bipolar meanings of age) were classified into three factors (evaluation, activity, and potency). Of the Japanese real words, 34 were associated with negative or positive features, 28 with passive or active features, and 35 with weak or strong features (Osgood and Suci 1955; Osgood et al. 1957; Staats and Staats 1957; Staats et al. 1959). The part of speech (word class) of some words used in the previous and current studies differs. Appendix A shows the full word list, including written Japanese words, the English meanings, written romaji (alphabetically written words for the pronunciations), the number of moras, and frequencies of the words shown in a Japanese psycholinguistic database (Amano and Kondo 2000; see Appendix A).

### Procedures

Participants judged what the presented words refer to and responded to a Google Form on their personal computers. They performed three types of judgments. First, as positiveness ratings, they evaluated whether the presented words had negative or positive features using a 5-point semantic differential scale from 1 (*negative*) to 5 (*positive*). Second, as activeness ratings, they judged whether the presented words had passive or active features using a 5-point semantic differential scale from 1 (*passive*) to 5 (*active*). Third, as strongness ratings, they evaluated whether the presented words had weak or strong features using a 5-point semantic differential scale from 1 (*weak*) to 5 (*strong*).

### Analyses

To examine the reliability of words associated with negative, positive, passive, active, weak, or strong features, we calculated Cronbach’s alpha using IBM SPSS 26.

## Results

Cronbach’s alphas of negative (*α* = 0.91), positive (*α* = 0.79), passive (*α* = 0.90), and active words (*α* = 0.80) were greater than 0.70 (Cortina 1993); however, we could not detect the sufficient reliability of weak and strong words higher than 0.70 (Cortina 1993). Therefore, we decided to use 28 Japanese real words associated with negative and positive features for Experiment 1 (14 each) and 26 Japanese real words associated with passive and active features for Experiment 2 (13 each).

## Experiment 1

### Participants

There were 37 participants (21 females; *M*_*age*_ = 36.24; *SD*_*age*_ = 7.35); all of them were native Japanese speakers. They were recruited from a crowdsourcing company in Japan (CrowdWorks, Inc.). The participants received a monetary reward of 220 Japanese yen, including the incidentals.

### Experimental paradigm

Experiment 1 employed a 2 × 3 within-subject factorial design. The dependent variables were evaluative responses to Japanese pseudowords (1: *negative*; 7: *positive*), and the independent variables were time (1: before conditioning; 2: after conditioning) and conditions (1: conditioning to positive words; 2: conditioning to negative words; 3: conditioning to neutral words).

### Experimental devices

An IC recorder (ICD-SX1000) was used to prepare spoken Japanese real words. Additionally, we used an online platform for behavioral experiments (gorilla.sc) to record the participants’ responses.

### Stimuli

We used four written pseudowords selected from psycholinguistic research on pseudowords (Umemoto et al. 1955), namely, *wayu* (*ワユ*), *sohi* (*ソヒ*), *nuyo* (*ヌヨ*), and *rehe* (*レヘ*). The four Japanese written pseudowords were presented with katakana characters which are Japanese characters that encompass a consonant and vowel or a vowel only (Yamaguchi 2007). In this study, we only used katakana characters that consisted of a consonant and a vowel; pseudowords consist of two katakana characters (Umemoto et al. 1955).

Additionally, we used 28 spoken Japanese positive (e.g., love) and negative words (e.g., fear) based on the results of Survey 1 (Appendix B). The results of Survey 1 showed that the mean positiveness scores of selected positive words ranged from 4.30 to 4.85, whereas the mean positiveness scores of selected negative words ranged from 1.21 to 2.07 (see Appendix B). We also used 28 spoken Japanese neutral words (e.g., lectures) based on a previous study, in which the authors detected Japanese real words associated with neutral emotional features (Higami et al. 2015). Another previous study (Staats and Staats 1957) used content words (e.g., nouns and adjectives) as positive and negative words and function words (e.g., prepositions such as “with”) as neutral words. However, since the differences in lexical categories (content words vs. function words) might have affected the previous study’s results, we decided to use only content words for all the spoken Japanese real words. A female native Japanese speaker (the first author) uttered the spoken Japanese real words (positive, negative, and neutral words) for recoding. We recorded the spoken Japanese real words in a quiet room to control for environmental sounds and noise.

### Procedures

The experiment consisted of a first evaluation phase (an evaluation task before conditioning), a conditioning phase, and a second evaluation phase (an evaluation task after conditioning). The three phases combined were of approximately 15 min. This experimental flow was approximately based on a previous study (Staats et al. 1959) that decreased the number of trials from Staats and Staats (1957). In their research, Staats and colleagues asked participants to rate pseudowords after the conditioning phase by using one of the scales linked to evaluation (valence: ratings from *pleasant* to *unpleasant*), activity (arousal: ratings from *active* to *passive*), or potency (dominance: ratings from *strong* to *weak*) in their three experiments. However, the previous studies (Staats and Staats 1957; Staats et al. 1959) did not include the first evaluation phase used in this study. Before the experiment, we stated “Please set your keyboard return to half-width characters and numbers, because you need to use the half-width numbers for your answers. Please set up your computer to allow you to hear the audio. We recommend that the volume of the sound is at least half of what you can hear clearly. To repeat spoken sounds verbally, please conduct the experiment in an environment where you can speak up. After you complete the settings, please press the space key to proceed to the experiment.” In both the evaluation phases (before and after conditioning), participants evaluated emotional features to four written Japanese pseudowords, namely, *wayu* (*ワユ*), *sohi* (*ソヒ*), *nuyo* (*ヌヨ*), and *rehe* (*レヘ*) using a 7-point semantic differential scale from 1 (*negative*) to 7 (*positive*; Osgood et al. 1957; Staats and Staats 1957; Staats et al. 1959). In each evaluation phase, participants pressed a key from one to seven on their keyboards. In the evaluation phases, each pseudoword presentation depended on participants’ responses. Furthermore, each stimulus was followed by a cross mark ( +) shown for 400 ms, involving 100 ms pauses before and after the cross mark. Before the first and second evaluation phases, we showed stated: “Please rate how negative or positive you feel about the katakana words displayed on the screen by pressing the keys 1 (*negative*) to 7 (*positive*) on the keyboard.” As a trial in the conditioning phase, we presented a written Japanese pseudoword for 5000 ms followed by a fixation (a cross mark: +) for 1200 ms, including 100 ms pauses before and after the fixation, and a spoken Japanese word selected from Survey 1. After presenting each spoken Japanese word, we asked the participants to repeat it verbally once. After producing the spoken word once, they pressed the key at their own pace. Before the conditioning phase, we stated: “Please watch the katakana words that will appear on the screen carefully, and carefully listen to and repeat the Japanese words that will be auditorily presented. Please press the space key to advance to the next task.” These methods were approximately similar to that of previous studies (Staats and Staats 1957; Staats et al. 1959). In all the phases (a first evaluation, conditioning, and second evaluation phases), the stimulus color was black, while the background color was white.

We counterbalanced word lists of the Japanese pseudowords and spoken Japanese real words between participants to control the effects of stimuli (De Houwer et al. 2001), such as the sound symbolic effects of stimuli, which linguistic features (e.g., spoken sounds) nonarbitrarily connect to referentially perceptual and emotional features (e.g., Ando et al. 2021; Kambara and Umemura 2021; Lin et al. 2021; Namba and Kambara 2020). Furthermore, to counterbalance word stimuli, we separated the samples into two groups. In the first group, *wayu* (*ワユ*) was conditioned to negative words and *nuyo* (*ヌヨ*) to positive words. Conversely, in the second group, *wayu* (*ワユ*) was conditioned to positive words and *nuyo* (*ヌヨ*) to negative words. Similarly, *sohi* (*ソヒ*) and *rehe* (*レヘ*) were conditioned to neutral words. Additionally, we randomized the presentation order of stimuli on gorilla.sc between participants to control the effects of the presentation order of written pseudowords and spoken words (Francis and Ciocca 2003). In a future direction in a previous research, the authors emphasized the importance of controlling the presentation order of stimuli (Ando et al. 2021).

### Analyses

We conducted a cumulative link mixed model (Christensen 2019) of two fixed effects of phases (before conditioning (phase 1); after conditioning (phase 2)) and conditions (a pseudoword conditioned to positive words; a pseudoword conditioned to neutral words; and a pseudoword conditioned to negative words), two random effects of participants and words (Baayen et al. 2008), and one dependent variable (evaluative responses to pseudowords) using “ordinal” (Christensen 2019) and “emmeans” packages (Lenth 2022) in R (R Core Team 2022). We also used “Rmisc” to calculate the means and 95% confidence intervals (Hope 2022), “FSA” to calculate medians and first and third quartiles (Q1 and Q3; Ogle et al. 2023), and “ggplot2” to make a figure of results (Wickham 2016) in R (Mangiafico 2015; Szuba et al. 2022). First, we employed the maximal random structure model for the model selection (Barr et al. 2013). We further simplified the maximal random structure model. Regarding the model selection, likelihood ratio tests were performed for the cumulative link mixed models using the anova () fuction on R (e.g., Baayen 2008; Winter 2020). Lastly, we applied random (varying) intercepts by participants and words and random (varying) slopes for conditions by participants as random effects (e.g., Baayen et al. 2008; Grasso et al. 2022; Winter 2020).

## Results

**Fig. 1:**
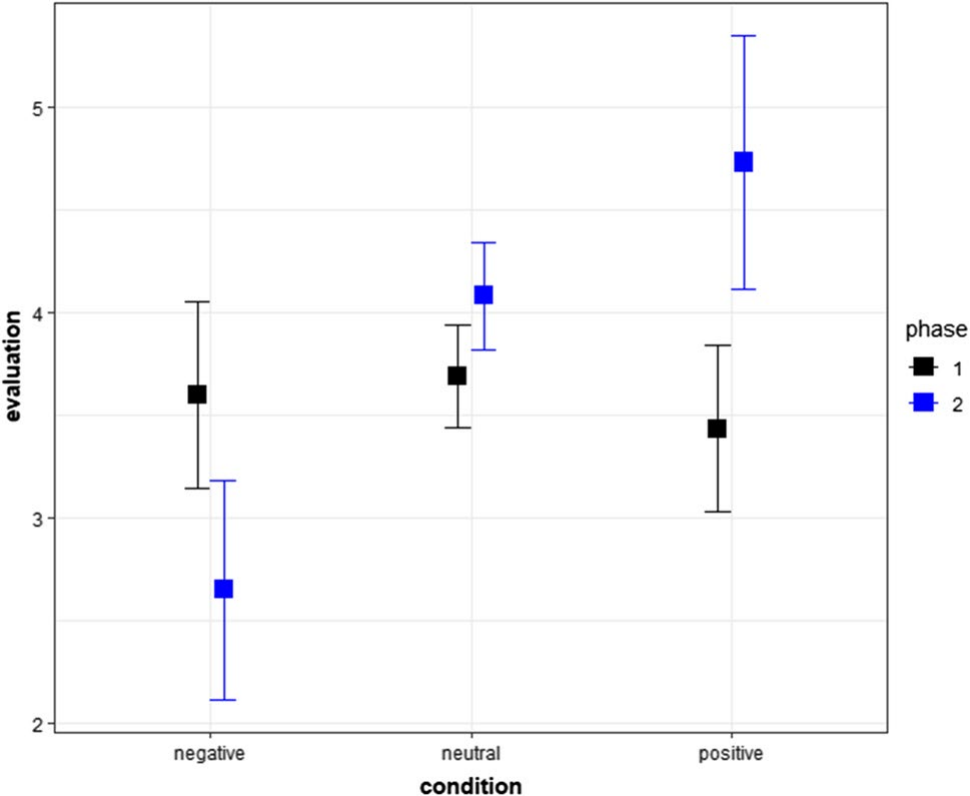
Means and 95% confidence intervals in Experiment 1. The values were calculated in an R package “Rmisc” (Hope 2022) on R (R Core Team 2022). We used “ggplot2” to create this figure (Wickham 2016) in R

In the cumulative link mixed model, we used raw evaluative responses of pseudowords (*nuyo*, *ヌヨ*; *wayu*, *ワユ*) conditioned to positive or negative features and raw evaluative responses of two pseudowords conditioned to neutral words (*sohi*, *ソヒ*; *rehe*, *レヘ*). The results showed a significant effect of phases and a significant interactions between phases and conditions (see Fig. 1 and Tables 1 and 2). The post-hoc tests showed that the evaluative responses of pseudowords conditioned to positive words were higher (more positive) after conditioning than before (Table 3). The evaluative responses of pseudowords conditioned to negative words were lower (more negative) after conditioning than before (Table 3). Additionally, the post-hoc tests showed that, after conditioning, evaluative responses of a pseudoword conditioned to positive and neutral words were higher (more positive) than those conditioned to negative words (Table 3). Table 1 shows medians and first and third quartiles (Q1 and Q3), whereas Fig. 1 shows the means and 95% confidence intervals.

**Table 1:** Medians and quartiles of evaluative responses in Experiment 1

Conditions	Before conditioning			After conditioning		
	*Mdn*	*Q1*	*Q3*	*Mdn*	*Q1*	*Q3*
Pseudoword conditioned to positive words	3.00	3.00	4.00	5.00	3.00	7.00
Pseudoword conditioned to negative words	4.00	2.00	4.00	2.00	1.00	4.00
Pseudoword conditioned to neutral words	4.00	3.00	4.75	4.00	3.25	5.00

*Mdn*: Medians; *Q1*: first quartiles; *Q3*: third quartiles. In Experiment 1, participants evaluated whether each pseudoword was positive or negative using a 7-point semantic differential scale ranging from 1 (*negative*) to 7 (*positive*). The *Mdns*, *Q1s*, and *Q3s* were calculated in an R package “FSA” (Ogle et al. 2023) on R (R Core Team 2022)

**Table 2:** Results of the cumulative link mixed model in Experiment 1

Random effects	Variance	*SD*	
Subjects (intercept)	3.09	1.76	
Condition Neutral	3.67	1.92	
Condition Positive	5.17	2.27	
Words (intercept)	0.05	0.22	

Fixed effects	Estimate	*SE*	*z* value
Phase 2***	− 1.71	0.47	− 3.67
Condition Neutral	0.20	0.53	0.38
Condition Positive	− 0.20	0.57	− 0.36
Phase 2: Condition Neutral***	2.32	0.56	4.15
Phase 2: Condition Positive***	3.84	0.68	5.66

***: *p* < .001; *SD*: standard deviation; *SE*: standard error. We used a package “ordinal” (Christensen 2019) on R (R Core Team 2022). Conditions consist of negative, neutral, and positive conditions. Phases consist of the first (before conditioning) and second phases (after conditioning)

**Table 3:** All the post-hoc tests of Experiment 1

Contrast	Estimate	*SE*	*z* ratio	*p* value
Phase 1 Negative—Phase 2 Negative**	1.71	0.47	3.67	0.0034
Phase 1 Negative—Phase 1 Neutral	− 0.20	0.53	− 0.38	0.999
Phase 1 Negative—Phase 2 Neutral	− 0.82	0.54	− 1.53	0.6468
Phase 1 Negative—Phase 1 Positive	0.20	0.57	0.36	0.9993
Phase 1 Negative—Phase 2 Positive*	− 1.93	0.61	− 3.17	0.0188
Phase 2 Negative—Phase 1 Neutral**	− 1.91	0.57	− 3.37	0.0099
Phase 2 Negative—Phase 2 Neutral***	− 2.52	0.58	− 4.39	0.0002
Phase 2 Negative—Phase 1 Positive	− 1.50	0.60	− 2.52	0.1189
Phase 2 Negative—Phase 2 Positive****	− 3.63	0.65	− 5.62	< .0001
Phase 1 Neutral—Phase 2 Neutral	− 0.62	0.30	− 2.06	0.3068
Phase 1 Neutral—Phase 1 Positive	0.40	0.53	0.76	0.9747
Phase 1 Neutral—Phase 2 Positive*	− 1.73	0.57	− 3.02	0.0306
Phase 2 Neutral—Phase 1 Positive	1.02	0.54	1.90	0.4036
Phase 2 Neutral—Phase 2 Positive	− 1.11	0.57	− 1.96	0.3653
Phase 1 Positive—Phase 2 Positive***	− 2.13	0.47	− 4.51	0.0001

****: *p* < .0001; ***: *p* < .001; **: *p* < .01; *: *p* < .05; + : .1 > *p* > .05. Conditions involve negative, neutral, and positive conditions. Phases involve the first (before conditioning) and second phases (after conditioning). We used the “emmeans” package (Lenth 2022) on R (R Core Team 2022). The comparisons were made using Tukey’s method

## Discussion

Experiment 1 investigated whether written Japanese pseudowords are conditioned to spoken Japanese positive or negative words. Two findings emerged: first, there was no significant difference among evaluative responses of conditions (evaluative responses of pseudowords conditioned to positive, negative, and neutral words) before conditioning; however, after conditioning, evaluative responses of pseudowords conditioned to positive and neutral words were higher than those conditioned to negative words. Second, after conditioning, pseudowords conditioned to positive words were higher (more positive) than before, while those conditioned to negative words were lower (more negative) than before. These findings are congruent to previous findings (e.g., Staats and Staats 1957). These results suggest that native Japanese speakers condition positive and negative evaluations of spoken Japanese words to those of written Japanese pseudowords. However, evaluative reponses of pseudowords conditioned to neutral words before and after conditioning differed insignificantly. This result indicates that the neutral words were functioned as neutral stimuli in Experiment 1. The experimental findings in Experiment 1 suggest that the evaluative responses of pseudowords conditioned to neutral words would not be associated with the mere-exposure effect or familiarity effect by which participants tend to prefer familiar things (Zajonc 1968, 2001; Monahan et al. 2000). The neutral words selected from a previous study (Higami et al. 2015) would be familiar to the participants. For example, words that people might use often (e.g., traffic light, home address, and road) are familiar words. Psycholinguistic studies have shown that the familiarity of verbal information positively correlates with the emotional valence (preference) of verbal information (e.g., Ando et al. 2021; Citron et al. 2014). In conjuction, these findings suggest that Japanese pseudowords could be conditioned to positive and negative words.

## Experiment 2

### Participants

There were 38 participants (17 females; *M*_*age*_ = 42.18; *SD*_*age*_ = 9.19); all of them were native Japanese speakers. They were recruited from a crowdsourcing company in Japan (CrowdWorks, Inc.). The participants received a monetary reward of 220 Japanese yen, including the incidentals.

### Experimental paradigm

Experiment 2 employed a 2 (before conditioning and after conditioning) × 3 (active, passive, and neutral) within-subjects factorial design. The dependent variables were evaluative responses to Japanese pseudowords (1: *passive*; 7: *active*), whereas the independent variables were time (1: before conditioning; 2: after conditioning) and conditions (1: conditioning to active words; 2: conditioning to passive words; 3: conditioning to neutral words).

### Experimental devices

An IC recorder (ICD-SX1000) was used to prepare spoken Japanese real words. Additionally, we used an online platform for behavioral experiments (gorilla.sc) to record the participants’ responses.

### Stimuli

We used four written pseudowords (*wayu*, *ワユ*; *sohi*, *ソヒ*; *nuyo*, *ヌヨ*; *rehe*, *レヘ*) that were the same as those used in Experiment 1.

Additionally, we used 26 spoken Japanese active (e.g., fast) and passive words (e.g., lazy) based on the results of Survey 1 (see Appendix C). The results of Survey 1 showed that the mean activeness scores of the selected active words ranged from 3.20 to 4.79, whereas the mean activeness scores of selected passive words ranged from 1.29 to 2.85 (see Appendix C). We also used 26 spoken Japanese real words associated with neutral features (e.g., lectures; Higami et al. 2015), which were the same as those used in Experiment 1, except for two words (area and staff; see Appendix D). A female native Japanese speaker (the first author) uttered the spoken Japanese real words for recoding. We recorded spoken Japanese real words in a quiet room to reduce environmental sounds and noise.

### Procedures

The methods of Experiment 2 were approximately the same as those used in Experiment 1. There were two procedural differences between Experiments 1 and 2. First, each written Japanese pseudoword (*nuyo* or *wayu*) was conditioned to 13 spoken Japanese active or passive words in Experiment 2, and to 14 spoken Japanese positive or negative words in Experiment 1. Second, in the first and second evaluation phases, participants in Experiment 2 evaluated emotional features to four written Japanese pseudowords consisting of *wayu* (ワユ), *sohi* (*ソヒ*), *nuyo* (*ヌヨ*), and *rehe* (*レヘ*) using a 7-point semantic differential scale from 1 (*passive*) to 7 (*active*), whereas participants in Experiment 1 evaluated them using a 7-point semantic differential scale from 1 (*negative*) to 7 (*positive*; Osgood et al. 1957; Staats and Staats 1957; Staats et al. 1959). Before the first and second evaluation phases in Experiment 2, we presented the following sentences: “Please rate how passive or active you feel about the katakana words displayed on the screen by pressing keys 1 (*passive*) to 7 (*active*) on the keyboard.” Additionally, we showed the following explanation before the conditioning phase in Experiment 2: “Please watch the katakana words that will appear on the screen carefully, and carefully listen to and repeat the Japanese words that will be auditorily presented. Press the space key to advance to the next task.”

### Analyses

We employed a cumulative link mixed model (Christensen 2019) of two fixed effects of phases and conditions, two random effects of participants and words (Baayen et al. 2008), and one dependent variable (evaluative responses to pseudowords) using “ordinal” (Christensen 2019) and “emmeans” packages (Lenth 2022) on R (R Core Team 2022). We also used “Rmisc” to calculate means and 95% confidence intervals (Hope 2022), “FSA” to calculate medians and first and third quartiles (Q1 and Q3; Ogle et al. 2023), and “ggplot2” to create a figure of results (Wickham 2016) as R packages (Mangiafico 2015; Szuba et al. 2022). Regarding the fixed effects, phases included before conditioning (phase 1) as 1 and after conditioning (phase 2) as 2, whereas conditions included a pseudoword conditioned to active words as active, a pseudoword conditioned to neutral words as neutral, and a pseudoword conditioned to passive words as passive. Regarding the model selection, we first applied the maximal random structure model (Barr et al. 2013). We further simplified the maximal random structure model. Regarding the model selection, likelihood ratio tests for the cumulative link mixed models were performed using the anova () fuction on R (e.g., Baayen 2008; Winter 2020). Lastly, we applied random (varying) intercepts by participants and words and random (varying) slopes for phases and conditions by participants as random effects (e.g., Baayen et al. 2008; Grasso et al. 2022; Winter 2020).

## Results

**Fig. 2:**
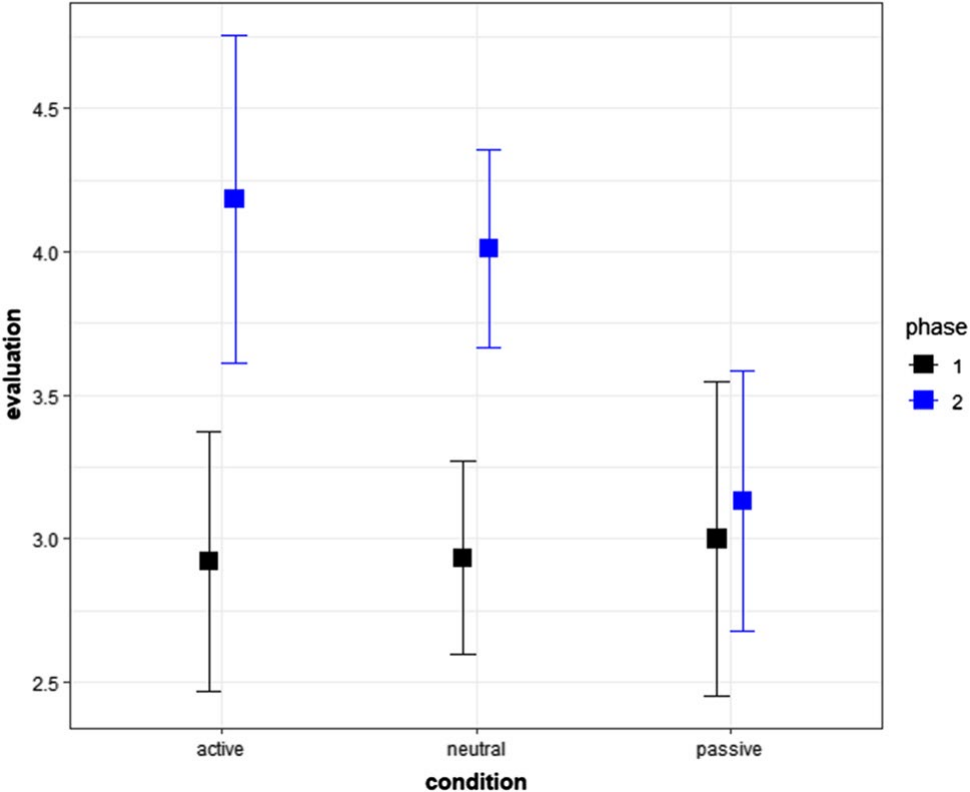
Means and 95% confidence intervals in Experiment 2. The values were calculated in an R package “Rmisc” (Hope 2022) on R (R Core Team 2022). We used “ggplot2” to create this figure (Wickham 2016) in R

In the cumulative link mixed model, we used raw evaluative responses of pseudowords (*nuyo*, *ヌヨ*; *wayu*, *ワユ*) conditioned to active or passive words and the raw evaluative responses of two pseudowords conditioned to neutral words (*sohi*, *ソヒ*; *rehe*, *レヘ*). The results showed that the effects of time and interaction between time and conditions were statistically significant, while that of conditions was not (Fig. 2 and Tables 4, 5, and 6). The post-hoc tests showed that the evaluative responses of pseudowords conditioned to active or neutral words were higher (more active) after conditioning than before. Additionally, the post-hoc analysis showed that, after conditioning, evaluative responses of a pseudoword conditioned to active words were moderately higher (more active) than those conditioned to passive words (close to being significantly different, *p* < 0.0551). Table 4 shows the medians and first and third quartiles (Q1 and Q3), whereas Fig. 2 shows the means and 95% confidence intervals.

**Table 4:** Medians and quartiles of evaluative responses in Experiment 2

Conditions	Before conditioning			After conditioning		
	*Mdn*	*Q1*	*Q3*	*Mdn*	*Q1*	*Q3*
Pseudoword conditioned to active words	3.00	2.00	4.00	4.00	3.00	5.75
Pseudoword conditioned to passive words	3.00	2.00	4.00	3.00	2.00	4.00
Pseudoword conditioned to neutral words	3.00	2.00	4.00	4.00	3.00	5.00

*Mdn*: Medians; *Q1*: first quartiles; *Q3*: third quartiles. In Experiment 2, participants evaluated whether each pseudoword was active or passive using a 7-point semantic differential scale from 1 (*passive*) to 7 (*active*). The *Mdns*, *Q1s,* and *Q3s* were calculated in an R package “FSA” (Ogle et al. 2023) on R (R Core Team 2022)

**Table 5:** Results of the cumulative link mixed model in Experiment 2

Random effects	Variance	*SD*	
Subjects (intercept)	2.37	1.54	
Phase2	2.09	1.45	
Condition Neutral	1.07	1.03	
Condition Passive	1.84	1.36	
Words (intercept)	0.06	0.24	

Fixed effects	Estimate	*SE*	*z* value
Phase 2***	1.81	0.50	3.59
Condition Neutral	− 0.07	0.47	− 0.14
Condition Passive	− 0.05	0.49	− 0.09
Phase 2: Condition Neutral	− 0.12	0.52	− 0.22
Phase 2: Condition Passive*	− 1.32	0.61	− 2.16

***: *p* < .001; * *p* < .05; *SD*: standard deviation; *SE*: standard error. We used a package “ordinal” (Christensen 2019) on R (R Core Team 2022). Conditions encompass active, neutral, and passive conditions. Phases encompass the first (before conditioning) and second phases (after conditioning)

**Table 6:** All the post-hoc tests of Experiment 2

Contrast	Estimate	*SE*	*z* ratio	*p value*
Phase 1 Active—Phase 2 Active**	− 1.81	0.50	− 3.59	0.0045
Phase 1 Active—Phase 1 Neutral	0.07	0.47	0.14	1.0000
Phase 1 Active—Phase 2 Neutral*	− 1.62	0.55	− 2.96	0.0362
Phase 1 Active—Phase 1 Passive	0.05	0.49	0.09	1.0000
Phase 1 Active—Phase 2 Passive	− 0.44	0.52	− 0.84	0.9600
Phase 2 Active—Phase 1 Neutral**	1.87	0.53	3.50	0.0061
Phase 2 Active—Phase 2 Neutral	0.18	0.47	0.39	0.9989
Phase 2 Active—Phase 1 Passive*	1.85	0.57	3.23	0.0159
Phase 2 Active—Phase 2 Passive +	1.37	0.49	2.82	0.0551
Phase 1 Neutral—Phase 2 Neutral***	− 1.69	0.40	− 4.28	0.0003
Phase 1 Neutral—Phase 1 Passive	− 0.02	0.56	− 0.04	1.0000
Phase 1 Neutral—Phase 2 Passive	− 0.50	0.58	− 0.87	0.9530
Phase 2 Neutral—Phase 1 Passive +	1.67	0.64	2.62	0.0927
Phase 2 Neutral—Phase 2 Passive	1.19	0.55	2.15	0.2606
Phase 1 Passive—Phase 2 Passive	− 0.48	0.50	− 0.98	0.9253

****: *p* < .0001; ***: *p* < .001; **: *p* < .01; *: *p* < .05; + : .1 > *p* > .05. Conditions involve active, neutral, and passive conditions. Phases involve the first (before conditioning) and second phases (after conditioning). We used the “emmeans” package (Lenth 2022) on R (R Core Team 2022). The comparisons were made using Tukey’s method

## Discussion

Experiment 2 examined whether written Japanese pseudowords are conditioned to spoken Japanese active or passive words. Two findings emerged: first, there was no significant difference among evaluative responses of conditions (evaluative responses of pseudowords conditioned to active, passive, and neutral words) before conditioning; however, after conditioning, evaluative responses of pseudowords conditioned to active words were moderately higher (more active) than those conditioned to passive words (close to being significantly different, *p* < 0.0551). Second, evaluative responses of pseudowords conditioned to active or neutral words were higher (more active) after conditioning than before, while there was no significant difference between the evaluative responses of pseudowords conditioned to passive words before and after conditioning. The evaluative responses of pseudowords conditioned to neutral words also increased after conditioning. The results of Experiment 2 suggest that the evaluative responses (activeness ratings) of a pseudoword conditioned to active words increase in the evaluative conditioning.

The neutral words used in this experiment might include words (e.g., road, lecture, and traffic light) that participants perceive as active, although we selected neutral words from a previous study that only identified emotional valence (associated with positiveness ratings in this study) and arousal (associated with activeness ratings in this study) of Japanese words (Higami et al. 2015). The neutral words selected from a previous study were specified as neutral using a scale associated with emotional valence (Higami et al. 2015). Regarding the final step of this study, we conducted a post-hoc survey (Survey 2) to examine the positiveness (positive/ negative) and activeness (active/ passive) ratings of all the words used in Experiments 1 and 2.

### Survey 2

### Participants

There were 30 participants (11 females; *M*_*age*_ = 43.33; *SD*_*age*_ = 8.39), all of which were native Japanese speakers, recruited from a crowdsourcing company in Japan (CrowdWorks, Inc.). The participants received a monetary reward of 165 Japanese yen, including the incidentals.

### Stimuli

The word stimuli was the only word used in Experiments 1 and 2 (see Appendix E, F, G, and H).

### Procedures

Participants determined what the presented words refer to and responded to a Google Form on their personal computers. They performed two types of judgments. In the first section, they evaluated whether the presented words had negative or positive features using a 7-point semantic differential scale ranging from 1 (*negative*) to 7 (*positive*). In the second section, they determined whether the presented words had passive or active features using a 7-point semantic differential scale ranging from 1 (*passive*) to 7 (*active*). The presentation order of the words was randomized in each section.

### Analyses

First, to check the positiveness of the words used in Experiment 1, we employed cumulative link mixed models including conditions (positive words, neutral words, and negative words) as fixed effects, participants and words as the random effects, and the ratings of positiveness as one dependent variable. Second, regarding the activeness of words in Experiment 1, we employed cumulative link mixed models including conditions (positive words, neutral words, and negative words) as fixed effects, participants and words as the random effects, and activeness ratings as one dependent variable. Third, regarding the positiveness of words in Experiment 2, we applied cumulative link mixed models including conditions (active words, neutral words, and passive words) as fixed effects, participants and words as the random effects, and positiveness ratings as one dependent variable. Fourth, to check the activeness of words used in Experiment 2, we employed cumulative link mixed models including conditions (active words, neutral words, and passive words) as fixed effects, participants and words as the random effects, and the ratings of activeness as one dependent variable. Regarding the cumulative link mixed models, we used “ordinal” (Christensen 2019) and “emmeans” packages (Lenth 2022) in R (R Core Team 2022). We also used “Rmisc” to calculate the means and 95% confidence intervals (Hope 2022), “FSA” to calculate medians and first and third quartiles (Q1 and Q3; Ogle et al. 2023), and “ggplot2” to create figures of results (Wickham 2016). First, we employed the maximal random structure model for the model selection (Barr et al. 2013). We further simplified the maximal random structure model. Regarding the model comparison, likelihood ratio tests were conducted for the cumulative link mixed models using the anova () fuction on R (e.g., Baayen 2008; Winter 2020). Lastly, we applied random (varying) intercepts by participants and words, and random (varying) slopes for conditions by participants as random effects in all the cumulative link mixed models (e.g., Baayen et al. 2008; Grasso et al. 2022; Winter 2020).

### Results of Survey 2: positiveness of words used in Experiment 1

**Fig. 3:**
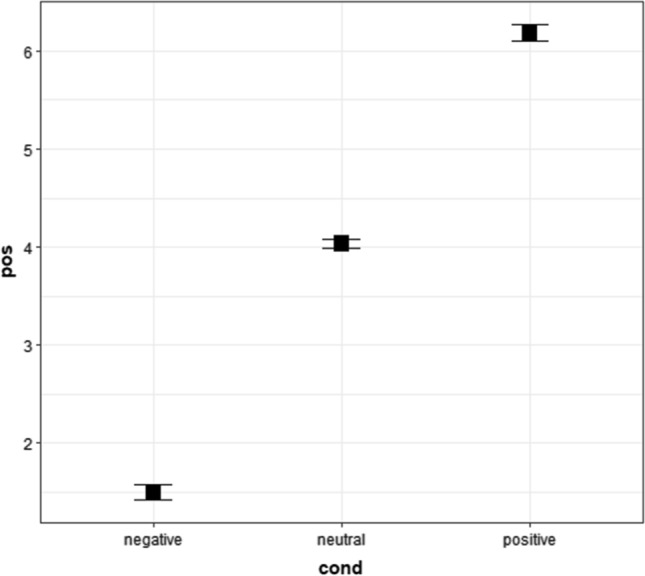
Means and 95% confidence intervals of positiveness ratings of words for Experiment 1 in Survey 2. “pos” means positiveness ratings from 1 (*negative*) to 7 (*positive*), whereas “cond” respresents conditions. The values were calculated in an R package “Rmisc” (Hope 2022) on R (R Core Team 2022). We used “ggplot2” to create this figure (Wickham 2016) in R

In the cumulative link mixed model, we used raw positiveness ratings of positive, neutral, and negative words in Survey 2. The results showed that the main effects of conditions were statistically significant (Fig. 3 and Tables 7, 8, and 9). The post-hoc tests showed that the positiveness ratings of positive words were higher (more positive) than those of neutral and negative words. Additionally, the post-hoc analysis showed that positiveness responses of neutral words were higher (more positive) than those of negative words. Table 7 shows the medians and first and third quartiles (Q1 and Q3), whereas Fig. 3 shows the means and 95% confidence intervals.

**Table 7:** Medians and quartiles of positiveness responses in Survey 2 for examining words used in Experiment 1

Conditions	*Mdn*	*Q1*	*Q3*
Positive words	6.00	6.00	7.00
Negative words	1.00	1.00	2.00
Neutral words	4.00	4.00	4.00

*Mdn*: Medians; *Q1*: first quartiles; *Q3*: third quartiles. In Survey 2, participants evaluated whether each word was positive or negative using a 7-point semantic differential scale ranging from 1 (*negative*) to 7 (*positive*). The *Mdns*, *Q1s,* and *Q3s* were calculated in an R package “FSA” (Ogle et al. 2023) on R (R Core Team 2022)

**Table 8:** Results of the cumulative link mixed model in Survey 2 for examining positiveness responses of words used in Experiment 1

Random effects	Variance	*SD*	
Words (intercept)	1.02	1.01	
Subjects (intercept)	4.15	2.04	
Condition Neutral	4.51	2.12	
Condition Positive	13.26	3.64	

Fixed effects	Estimate	*SE*	*z* value
Condition Neutral***	8.95	0.65	13.75
Condition Positive***	15.63	0.92	17.02

***: *p* < .001; * *p* < .05; *SD*: standard deviation; *SE*: standard error. We used a package “ordinal” (Christensen 2019) on R (R Core Team 2022). Conditions consist of positive, neutral, and negative conditions

**Table 9:** All the post-hoc tests of Survey 2 for examining positiveness responses of words used in Experiment 1

Contrast	Estimate	*SE*	*z* ratio	*p value*
Negative – Neutral****	− 8.95	0.65	− 13.75	< .0001
Negative –Positive****	− 15.63	0.92	− 17.02	< .0001
Neutral –Positive****	− 6.68	0.54	− 12.44	< .0001

****: *p* < .0001; ***: *p* < .001; **: *p* < .01; *: *p* < .05; + : .1 > *p* > .05. Conditions involve positive, neutral, and negative conditions. We used the “emmeans” package (Lenth 2022) on R (R Core Team 2022). The comparisons were made using Tukey’s method

### Results of Survey 2: activeness of words used in Experiment 1

**Fig. 4:**
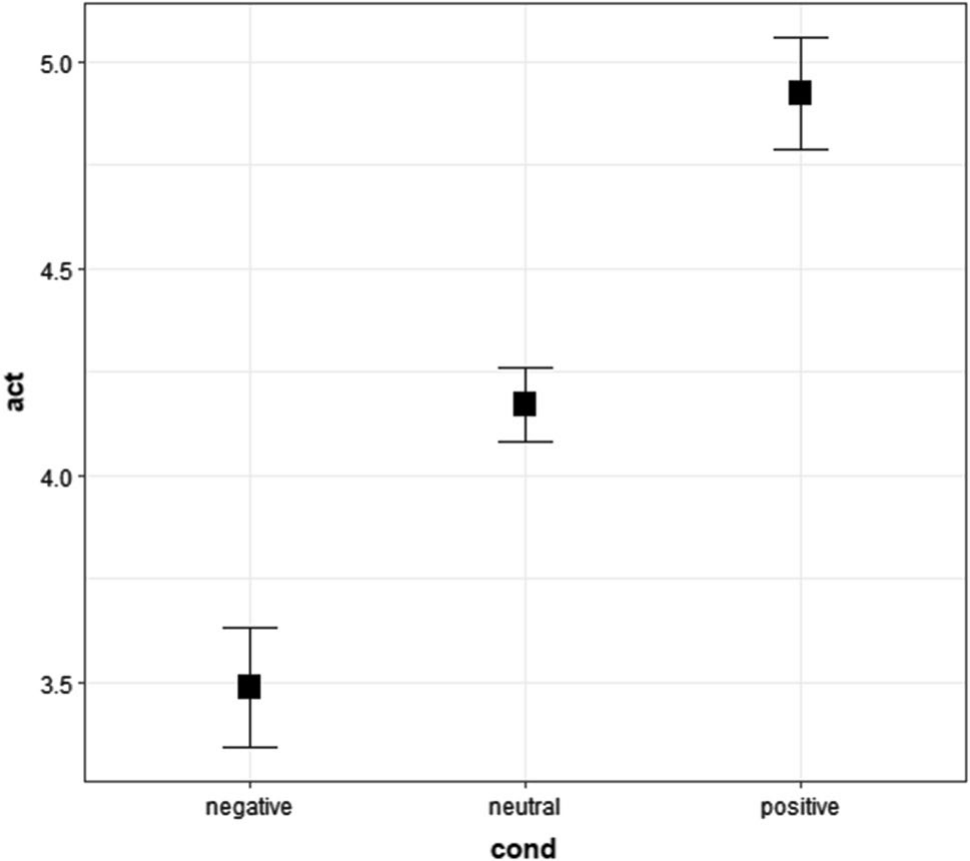
Means and 95% confidence intervals of activeness ratings of words for Experiment 1 in Survey 2. “act” represents activeness ratings from 1 (*passive*) to 7 (*active*), whereas “cond” represents conditions. The values were calculated in an R package “Rmisc” (Hope 2022) on R (R Core Team 2022). We used “ggplot2” to create this figure (Wickham 2016) in R

In the cumulative link mixed model, we used raw activeness ratings of positive, neutral, and negative words in Survey 2. The results showed that the main effects of conditions were statistically significant (Fig. 4 and Tables 10, 11, and 12). The post-hoc tests showed that the activeness ratings of positive words were higher (more active) than those of neutral and negative words. Additionally, the post-hoc analysis showed that the activeness responses of neutral words were higher (more active) than those of negative words. Table 10 shows the medians and first and third quartiles (Q1 and Q3), whereas Fig. 4 shows the means and 95% confidence intervals.

**Table 10:** Medians and quartiles of activeness responses in Survey 2 for examining words used in Experiment 1

Conditions	*Mdn*	*Q1*	*Q3*
Positive words	5.00	4.00	6.00
Negative words	3.00	2.00	5.00
Neutral words	4.00	4.00	5.00

*Mdn*: Medians; *Q1*: first quartiles; *Q3*: third quartiles. In Survey 2, participants evaluated whether each word was active or passive using a 7-point semantic differential scale ranging from 1 (*passive*) to 7 (*active*). The *Mdns*, *Q1s,* and *Q3s* were calculated in an R package “FSA” (Ogle et al. 2023) on R (R Core Team 2022)

**Table 11:** Results of the cumulative link mixed model in Survey 2 for examining activeness responses of words used in Experiment 1

Random effects	Variance	*SD*	
Words (intercept)	1.31	1.15	
Subjects (intercept)	2.09	1.45	
Condition Neutral	2.46	1.57	
Condition Positive	4.12	2.03	

Fixed effects	Estimate	*SE*	*z* value
Condition Neutral*	1.22	0.49	2.52
Condition Positive***	2.74	0.59	4.66

***: *p* < .001; * *p* < .05; *SD*: standard deviation; *SE*: standard error. We used a package “ordinal” (Christensen 2019) on R (R Core Team 2022). Conditions encompass positive, neutral, and negative conditions

**Table 12:** All the post-hoc tests of Survey 2 for examining activeness responses of words used in Experiment 1

Contrast	Estimate	*SE*	*z* ratio	*p value*
Negative—Neutral*	− 1.22	0.49	− 2.52	0.0315
Negative—Positive****	− 2.74	0.59	− 4.66	< .0001
Neutral—Positive**	− 1.51	0.44	− 3.42	0.0018

****: *p* < .0001; ***: *p* < .001; **: *p* < .01; *: *p* < .05; + : .1 > *p* > .05. Conditions encompass positive, neutral, and negative conditions. We used the “emmeans” package (Lenth 2022) on R (R Core Team 2022). The comparisons were made using Tukey’s method

### Results of Survey 2: positiveness of words used in Experiment 2

**Fig. 5:**
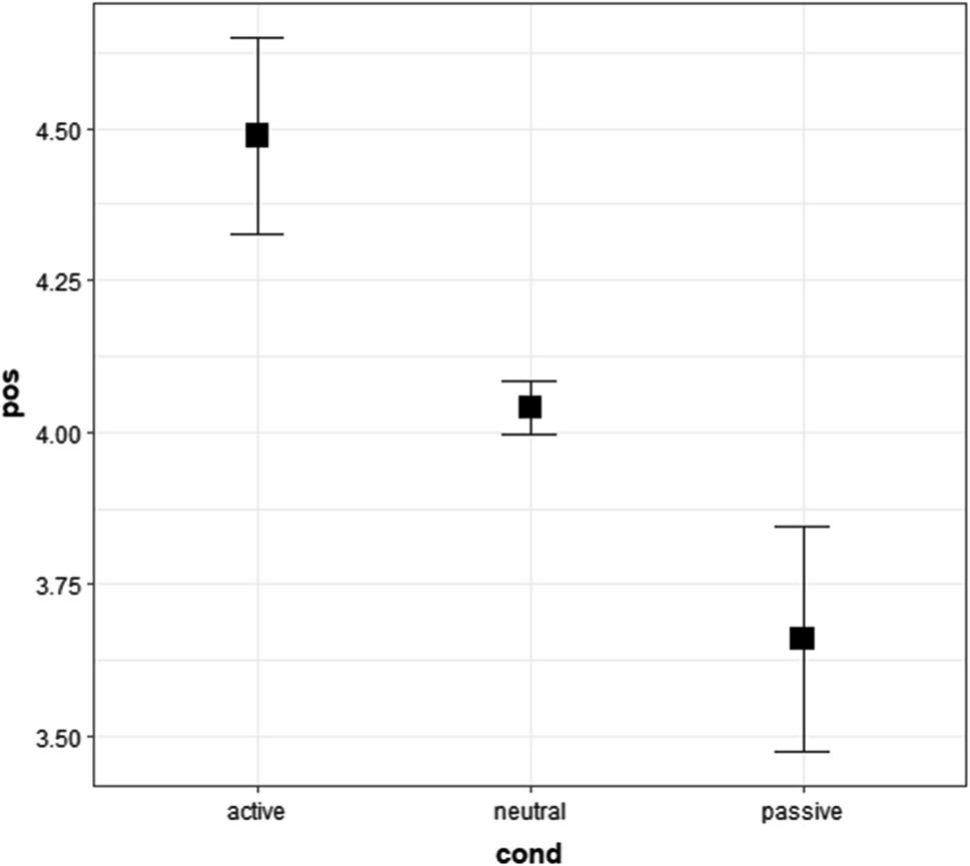
Means and 95% confidence intervals of positiveness ratings of words for Experiment 2 in Survey 2. “pos” represents positiveness ratings from 1 (*negative*) to 7 (*positive*), whereas “cond” represents conditions. The values were calculated in an R package “Rmisc” (Hope 2022) on R (R Core Team 2022). We used “ggplot2” to create this figure (Wickham 2016) in R

In the cumulative link mixed model, we used raw positiveness ratings of active, neutral, and passive words in Survey 2. The results showed that the main effects of conditions were moderately significant (close to being significant difference, *p* < 0.0508); Fig. 5 and Tables 13, 14, and 15). The post-hoc tests, which were employed to check differences among conditions, showed that the positiveness ratings of active, neutral, and passive words differed insignificantly. Table 13 shows the medians and first, and third quartiles (Q1 and Q3), whereas Fig. 5 shows the means and 95% confidence intervals.

**Table 13:** Medians and quartiles of positiveness responses in Survey 2 for examining words used in Experiment 2

Conditions	*Mdn*	*Q1*	*Q3*
Active words	5.00	3.00	6.00
Passive words	3.00	2.00	5.00
Neutral words	4.00	4.00	4.00

*Mdn*: Medians; *Q1*: first quartiles; *Q3*: third quartiles. In Survey 2, participants evaluated whether each word was positive or negative using a 7-point semantic differential scale ranging from 1 (*negative*) to 7 (*positive*). The *Mdns*, *Q1s,* and *Q3s* were calculated in an R package “FSA” (Ogle et al. 2023) on R (R Core Team 2022)

**Table 14:** Results of the cumulative link mixed model in Survey 2 for examining positiveness responses of words used in Experiment 2

Random effects	Variance	*SD*	
Words (intercept)	7.88	2.81	
Subjects (intercept)	0.35	0.59	
Condition Neutral	0.74	0.86	
Condition Passive	1.09	1.04	

Fixed effects	Estimate	*SE*	*z* value
Condition Neutral	− 1.15	0.98	− 1.18
Condition Passive +	− 2.20	1.13	− 1.95

***: *p* < .001; **: *p* < .01; *: *p* < .05; + : .1 > *p* > .05; *SD*: standard deviation; *SE*: standard error. We used a package “ordinal” (Christensen 2019) on R (R Core Team 2022). Conditions encompass active, neutral, and passive conditions

**Table 15:** All the post-hoc tests of Survey 2 for examining positiveness responses of words used in Experiment 2

Contrast	Estimate	*SE*	*z* ratio	*p value*
Active—Neutral	1.15	0.98	1.18	0.4645
Active—Passive	2.20	1.13	1.95	0.1241
Neutral—Passive	1.05	0.97	1.08	0.5258

****: *p* < .0001; ***: *p* < .001; **: *p* < .01; *: *p* < .05; + : .1 > *p* > .05. Conditions involve active, neutral, and passive conditions. We used the “emmeans” package (Lenth 2022) on R (R Core Team 2022). The comparisons were made using Tukey’s method

### Results of Survey 2: activeness of words used in Experiment 2

**Fig. 6:**
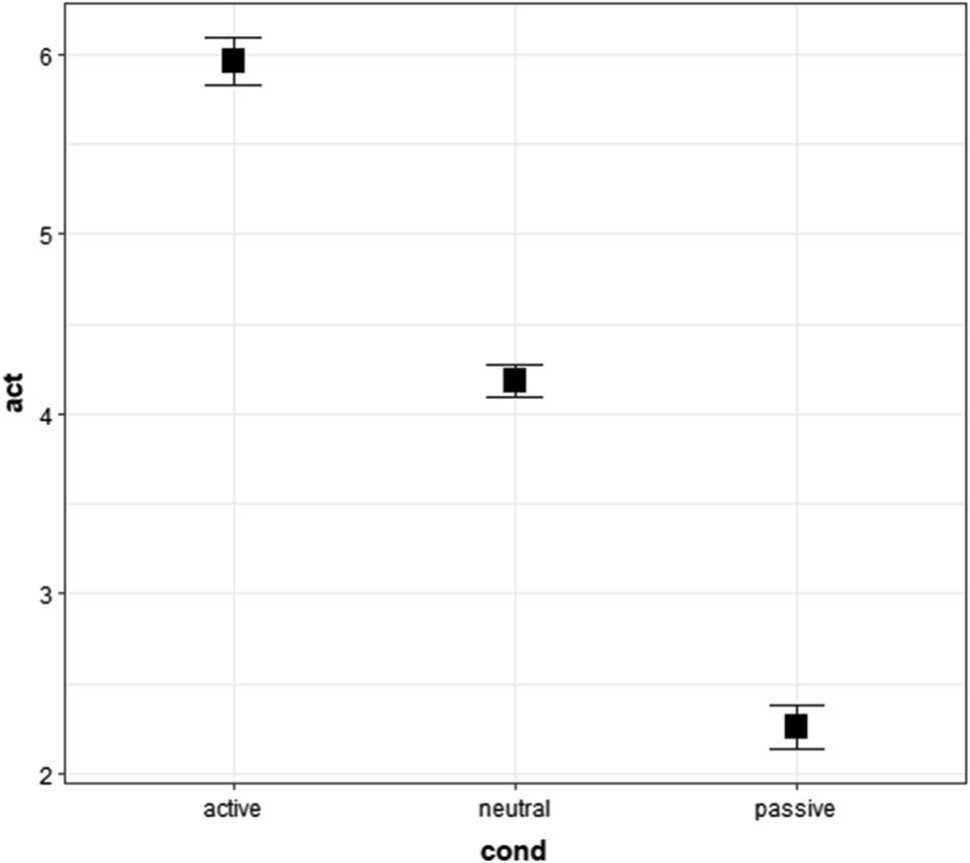
Means and 95% confidence intervals of activeness ratings of words for Experiment 2 in Survey 2. “act” represents activeness ratings from 1 (*passive*) to 7 (*active*), whereas “cond” represents conditions. The values were calculated in an R package “Rmisc” (Hope 2022) on R (R Core Team 2022). We used “ggplot2” to create this figure (Wickham 2016) in R

We used raw activeness ratings of active, neutral, and passive words in Survey 2 in the cumulative link mixed model. The results showed that the main effects of conditions were significant (Fig. 6 and Tables 16, 17, and 18). The post-hoc tests showed that the activeness ratings of active words were higher (more active) than those of neutral and passive words. Additionally, the post-hoc analysis showed that activeness responses of neutral words were higher (more active) than those of passive words. Table 16 shows the medians and first, and third quartiles (Q1 and Q3), whereas Fig. 6 shows the means and 95% confidence intervals.

**Table 16:** Medians and quartiles of activeness responses in Survey 2 for examining words used in Experiment 2

Conditions	*Mdn*	*Q1*	*Q3*
Active words	6.00	5.00	7.00
Passive words	2.00	1.00	3.00
Neutral words	4.00	4.00	5.00

*Mdn*: Medians; *Q1*: first quartiles; *Q3*: third quartiles. In Survey 2, participants evaluated whether each word was active or passive using a 7-point semantic differential scale ranging from 1 (*passive*) to 7 (*active*). The *Mdns*, *Q1s,* and *Q3s* were calculated in an R package “FSA” (Ogle et al. 2023) on R (R Core Team 2022)

**Table 17:** Results of the cumulative link mixed model in Survey 2 for examining activeness responses of words used in Experiment 2

Random effects	Variance	*SD*	
Words (intercept)	1.55	1.25	
Subjects (intercept)	2.33	1.53	
Condition Neutral	1.55	1.24	
Condition Passive	6.30	2.51	

Fixed effects	Estimate	*SE*	*z* value
Condition Neutral***	− 3.86	0.51	− 7.57
Condition Passive***	− 7.56	0.71	− 10.63

***: *p* < .001; * *p* < .05; *SD*: standard deviation; *SE*: standard error. We used a package “ordinal” (Christensen 2019) on R (R Core Team 2022). Conditions encompass active, neutral, and passive conditions

**Table 18:** All the post-hoc tests of Survey 2 for examining activeness responses of words used in Experiment 2

Contrast	Estimate	*SE*	*z* ratio	*p value*
Active—Neutral****	3.86	0.51	7.57	< .0001
Active—Passive****	7.56	0.71	10.63	< .0001
Neutral—Passive****	3.70	0.58	6.40	< .0001

****: *p* < .0001; ***: *p* < .001; **: *p* < .01; *: *p* < .05; + : .1 > *p* > .05. Conditions encompass active, neutral, and passive conditions. We used the “emmeans” package (Lenth 2022) on R (R Core Team 2022). The comparisons were made using Tukey’s method

## Discussion

In Survey 2, we examined the differences among positive and active responses of word conditions used in Experiments 1 and 2. Regarding the words used in Experiment 1, the results of Survey 2 showed that positiveness and activeness ratings of positive words were higher than those of neutral and negative words. Additionally, the positiveness and activeness ratings of neutral words were higher than those of negative words. Regarding the words used in Experiment 2, the results of Survey 2 showed that the activeness ratings of active words were higher than those of neutral and passive words. Additionally, the activeness ratings of neutral words were higher than those of passive words. However, the positiveness rating of words used in Experiment 2 differed insignificantly. The results of Survey 2 suggest that although the positiveness and activeness responses of words used in Experiment 1 and 2 were controlled, no significant difference among the positiveness responses of words might influence the evaluative conditioning in Experiment 2.

Regarding the evaluative conditioning, participants may interactively condition the positiveness and activeness ratings of words to those of pseudowords. A previous study showed that the positiveness (associated with valence in the previous study) and activeness ratings (associated with arousal in the previous study) were positively correlated in the effectively evaluative conditioning (Gawronski and Mitchell 2014). These results are consistent with our findings of Experiment 1 and Survey 2. Gawronski and Mitchell also showed that evaluative conditioning is more effective for high active UCS (associated with high arousal UCS in the previous study) than low active UCS (associated with high arousal UCS in the previous study; Gawronski and Mitchell 2014). Additionally, the previous study indicated that the activeness (arousal) ratings of stimuli may influence the memory of CS and UCS pairs (Gawronski and Mitchell 2014). However, Survey 2 showed that whereas the positiveness ratings of the words used in Experiment 2 differed insignificantly, only the activeness ratings of active words were higher than those of neutral and passive words. The results of Experiment 2 showed that participants conditioned the activeness ratings of active words to those of pseudowords, while they did not condition those of passive words to those of pseudowords. In sum, this finding suggests that when participants condition activeness (arousal) ratings of passive (low arousal) words to those of pseudowords, the positiveness (valence) ratings of words would be important in the evaluative conditioning.

### General discussion

In this study, we conducted two behavioral experiments after selecting the stimuli in a survey study. The findings of Experiment 1 suggest that evaluative responses of written Japanese pseudowords could be conditioned to spoken Japanese positive or negative words, while the conditioning might not be affected by the mere-exposure effect or familiarity effect of verbal information (Ando et al. 2021; Citron et al. 2014; Monahan et al. 2000; Zajonc 1968, 2001). Similarly, the results of Experiment 2 suggest that the evaluative responses of written Japanese pseudowords could also be conditioned to spoken Japanese active words. In future, we propose the following methods: first, we selected the neutral words used as a control condition when conditioning evaluative responses of pseudowords to active and passive words. However, these words might not be neutral for the participants in Experiment 2 because the neutral words selected from a previous study were specified as neutral using a scale associated with emotional valence (Higami et al. 2015). Regarding checking the positiveness and activeness ratings of the words used in Experiment 2, the results of the post-hoc survey (Survey 2) suggest that neutral words functioned as neutral words, whereas the positiveness ratings of active, passive, and neutral words used in Experiment 2 differed insignificantly. Therefore, the results of Survey 2 suggest that when participants condition the activeness ratings of passive words to those of pseudowords, the positiveness ratings of words would be essential to effective evaluative conditioning. Second, future studies can replicate this study, which measured evaluative responses before and after conditioning using English stimuli and asking native English speakers since Staats and Staats (1957) and Staats et al. (1959) only focused on the evaluative responses after conditioning. Third, although the current and previous studies used spoken real words as UCS and written pseudowords as CS, researchers can use them conversely. If future studies also use written real words as UCS and spoken pseudowords as CS in their experiments, researchers can examine the differential effects of the stimulus modalities in the verbally evaluative conditioning. Finally, since conditioning evaluative responses of written pseudowords to positive and active words were robust for participants, there is scope to examine the clinical application of this approach for patients in future. If the vocabulary of patients can be conditioned to positive or active words, the evaluative responses of these words stored in their vocabulary may increase.

In this study, we improved some experimental methods of previous studies, which conducted verbally evaluative conditioning (Passalli et al. 2022; Staats and Staats 1957; Staats et al. 1959; Vidal et al. 2021). First, although they could not examine differences between subjective evaluations of pseudowords before and after conditioning subjective evaluations (meanings) of spoken real words to those of pseudowords (Staats and Staats 1957), we assessed the differences between them. Second, since few studies had examined whether native speakers of non-alphabetic languages can condition the subjective evaluations (meanings) of the non-alphabetic real words to those of the non-alphabetic pseudowords, we examined whether Japanese native speakers can condition subjective evaluations (meanings) of Japanese real words to those of Japanese pseudowords. This is the first study to show the effects of verbal evaluative conditioning in Japanese.

We use the experimental procedures (the conditioning phase and evaluation phase after the conditioning phase) of Staats and colleagues’ research (e.g., Staats and Staats 1957; Staats et al. 1959). In the conditioning phase, participants explicitly repeated the spoken CS by speech production. For their speech productions, the participants listened to, kept, and repeated each spoken stimulus. This procedure would be associated with working memory processes for the maintenance and scanning of verbal stimuli (e.g., Kambara et al. 2017, 2018). The performance of a working memory task would be correlated with the performance of the associative learning for linguistic and referential features (e.g., Horinouchi et al. under review). If speech production was not included in this study, participants may have not paid attention to the spoken CS. If so, the effects of the verbally evaluative conditioning may be weak. To check the attentional and learning effects in verbally evaluative conditioning, a related study used a memory task (e.g., Vidal et al. 2021). Future studies can examine the relationships between the effects and procedural differences in verbally evaluative conditioning.

Future studies should consider the following points based on our study’s limitations: first, future research needs to consider the selection of pseudowords. The four pseudowords used in Experiments 1 and 2 were selected from a Japanese psycholinguistic study (Umemoto et al. 1955), despite the associative and meaningful values of these pseudowords being extremely low in the previous study. Therefore, all the emotional dimensions should be checked before future studies because the values of these pseudowords may change in future studies in light of increased proficiency of second languages, neologisms, and borrowings among others. For example, a Japanese person may think that a pseudoword (*nuyo*: *ヌヨ*) is orthographically similar to another word (*mayo*: *マヨ*), which means mayonnaise in Japanese.

Second, future studies should use the same scales in the survey and experiments. In our preliminary survey (Survey 1), we used five-point scales. In the two experiments and post-hoc survey (Survey 2), we used seven-point scales. The number of points in these scales may affect the evaluations in studies (e.g., Dawes 2008).

Third, future studies should check the valence and arousal values of each word (UCS) and pseudoword (CS) if those studies use the current paradigm. In this study, we did not collect both valence and arousal evaluations of each word and pseudoword before the experiments. We can check not only the participants’ ratings of pseudowords but also those of the real words in the first evaluation phase. Accordingly, we can use those ratings to create custom positive, neutral, and negative words for each participant (e.g., a person would think that “lecture” is a very negative word). Moreover, the neutral words should be determined based on similar samples and scales (positive–negative, active–passive, and strong–weak scales). Hence, researchers need to ensure that the Japanese psycholinguistic database includes the valence and arousal evaluations of many words.

Fourth, to the best of our knowledge, although previous studies have investigated the longitudinal effects of associative learning for linguistic and nonlinguistic features (e.g., Havas et al. 2018; Kambara et al. 2013; Lee et al. 2003; Takashima et al. 2017), no study has examined the longitudinal effects of verbally evaluative conditioning. Some studies longitudinally showed the evaluative conditioning effects of pictures days after the evaluative conditioning (e.g., Förderer and Unkelbach 2013; Waroquier et al. 2020). A related study suggested that evaluative conditioning effects were not affected by sleep connected to memory consolidation (Richter et al. 2021). Because the evaluative conditioning effects of pictures remained after the conditioning day, the effects of verbally evaluative conditioning would also be maintained.

Fifth, we only specified the participants as native Japanese speakers when we recruited participants in this study. We did not collect the linguistic experience of the participants (e.g., their experiences of L2 learning). In Japan, most people generally learn English from their elementary or junior high school through university. Because the experiences of L2 learning may influence verbally evaluative conditioning, future studies may consider the experiences of language learning. A previous study showed that the effects of verbally evaluative conditioning in L1 were greater than those in L2 (Vidal et al. 2021).

Lastly, researchers should consider CS, UCS, and other experimental methods in evaluative conditioning. In a review article, De Houwer et al. (2001) reported that evaluative conditioning is robust and ubiquitous. However, some failures of evaluative conditioning occur owing to the methodological variety, stimuli, number of stimuli, order of stimulus presentation, and experimental design. In evaluative conditioning, the identification of CS would be an important factor (Stahl and Bading 2020; Stahl et al. 2016). Moreover, foveal presentation of CS (photographs) would also be more effective than parafoveal presentation of CS in the evaluative conditioning of photographs as CS and emotional faces as UCS (Dedonder et al. 2014). The effects of evaluative conditioning are higher for simultaneous pairings than for sequential pairings, because attention and memory affect the evaluative conditioning (Stahl and Heycke 2016). Heycke and colleagues reported that the effects of evaluative conditioning were cross-modally greater for visual CSs with 1000 ms than those with fast presentation when auditory UCSs were used (Heycke et al. 2017). The evaluative conditioning of affective pictures (UCS) and verbal stimuli (CS) supraliminally appeared in artificial grammers (Jurchiş et al. 2020) and an unfamiliar language (Amd 2022). Additionally, Kuchinke et al. (2015) reported that early modulations of event-related potentials occur during the recognition of pseudowords conditioned to emotional pictures. In consideration of the methods, researchers also need to consider samples for experiment. Regarding the development perspectives of the evaluative conditioning, the evaluative conditioning has been shown for preschool- and school-aged children (Field 2006; Halbeisen et al. 2017, 2021). However, a related study showed no effect of the evaluative conditioning including both of the verbal stimuli and nonverbal stimuli for school-aged children (Charlesworth et al. 2020). These previous findings imply that nonverbal stimuli might be more effective than verbal stimuli in the evaluative conditioning, especially for early aged children because the early aged children would have lower repertories of words (associative pairs of linguistic and nonlinguistic information) in their mental lexicon than adults.

Regarding the implications of this study in the real world, verbally evaluative conditioning can be applied as a psycholinguistic therapy. For example, if a person has fearful or negative emotions associated with a word (e.g., a person name, a specific word like school), verbally evaluative conditioning can be useful to improve the evaluation of the word. Future studies can employ this perspective in applied studies.

In conclusion, this study identifies the verbally evaluative conditioning of Japanese words connected to negative, positive, and active emotions for Japanese native speakers. We improved the methods used in previous studies. We also discuss the future directions and limitations of our study for future research.

## Data Availability

The analyzed data and spoken stimuli are available on Open Science Framework (https://osf.io/re2s4/?view_only=8543196d6e9c4aa8a1e2b4f2ed7ceed9).

## Appendix A Words in Survey 1

**Table:**

Category	Japanese word	English word	Japanese word in romaji	Mora	Frequency
Positive	美しい	Beautiful +	utsukushī	5	8048
Positive	勝つ	Win	katsu	2	7886
Positive	贈り物	Gift	okurimono	5	1337
Positive	甘い	Sweet	amai	3	6550
Positive	正直	Honesty*	shōjiki	4	829
Positive	賢い	Smart	kashikoi	4	634
Positive	豊か	Rich	yutaka	3	1163
Positive	神聖	Sacredness*	shinsei	4	200
Positive	友達	Friend	tomodachi	4	5045
Positive	尊い	Valuable	tōtoi	4	418
Positive	幸せ	Happiness*	shiawase	4	2738
Positive	可愛い	Pretty	kawaii	4	399
Positive	健康	Health*	kenkō	4	16,700
Positive	成功	Success	seikō	4	23,145
Positive	金	Money	kane	2	–
Positive	休暇	Vacation	kyūka	3	4669
Positive	愛	Love	ai	2	6404
Negative	泥棒	Thief	dorobō	4	758
Negative	苦い	Bitter	nigai	3	2117
Negative	醜い	Ugly	minikui	4	384
Negative	悲しい	Sad	kanashī	4	3725
Negative	価値がない	Worthless	kachiganai	5	–
Negative	酸っぱい	Sour	suppai	4	140
Negative	残酷	Cruelty*	zankoku	4	273
Negative	敵	Enemy	teki	2	4487
Negative	汚い	Dirty	kitanai	4	1091
Negative	邪悪	Evil	jaaku	3	38
Negative	病気	Sickness*	byōki	3	16,426
Negative	愚かな	Stupid	orokana	4	–
Negative	失敗	Failure	shippai	4	14,314
Negative	嫌悪	Disgust*	ken’o	3	527
Negative	恐れ	Fear	osore	3	16,853
Negative	狂気	Insanity*	kyōki	3	547
Negative	毒	Poison	doku	2	1700
Active	はやい (早い・速い)	Fast/quick	hayai	3	24,004
Active	猛烈	Ferocity*	mōretsu	4	162
Active	緊張	Tension*	kinchō	4	13,688
Active	元気	Spryness*	genki	3	4352
Active	熱い	Hot	atsui	3	4362
Active	活発	Briskness*	kappatsu	4	535
Active	かき乱す (搔き乱す)	Agitate	kakimidasu	5	65
Active	熱心な	Eager	nesshinna	5	–
Active	鋭い	Sharp	surudoi	4	4166
Active	急ぐ	Hasten +	isogu	3	14,479
Active	興奮	Excitement*	kōfun	4	2998
Active	若い	Young	wakai	3	25,019
Active	急かす (せかす)	Hustle	sekasu	3	204
Passive	ぶらつく	Loafing	buratsuku	4	54
Passive	微睡み (まどろみ)	Slumber	madoromi	4	21
Passive	落ち着いた	Cool/relaxed	ochitsuita	5	–
Passive	気だるい	Listless	kedarui	4	14
Passive	眠い	Drowsy	nemui	3	393
Passive	鈍い	Dull	nibui	3	2119
Passive	怠惰	Laziness*	taida	3	85
Passive	穏やか	Calm	odayaka	4	305
Passive	お年寄り	Old	otoshiyori	5	–
Passive	遅い	Slow	osoi	3	9100
Passive	安心した	Relaxed	anshinshita	6	–
Passive	休憩	Resting	kyūkei	4	1453
Passive	不活性	Inertness*	fukassei	5	–
Passive	平和	Peace*	heiwa	3	38,372
Passive	虚しい	Lifeless	munashī	4	–
Strong	力強い	Powerful	chikarazuyoi	6	2439
Strong	がっしりとした	Robust	gasshiritoshita	7	–
Strong	頑丈	Sturdiness*	ganjō	4	61
Strong	男らしい	Masculine	otokorashī	6	188
Strong	逞しい (たくましい)	Athletic/rugged	takumashī	5	1223
Strong	健康的	Healthy	kenkōteki	6	–
Strong	重い	Heavy	omoi	3	15,520
Strong	険しい	Sharp	kewashī	4	1410
Strong	派手な	Loud	hadena	3	–
Strong	活動的	Active	katsudōteki	6	40
Strong	硬い	Hard	katai	3	3033
Strong	勇敢	Bravery*	yūkan	4	62
Strong	凄まじい (すさまじい)	Heavy	susamajī	5	1140
Strong	急激な	Sharp/rapid**	kyūgekina	5	–
Strong	豊か	Rich	yutaka	3	1163
Strong	幅広い	Wide	habahiroi	5	10,817
Strong	濃い	Thick	koi	2	6398
Strong	大きい	Large	ōkī	4	80,410
Weak	不自由	Cripple*	fujiyū	4	1117
Weak	希薄	Thinness*	kihaku	3	585
Weak	柔らかい	Soft	yawarakai	5	1319
Weak	脆い	Frail	moroi	3	57
Weak	狭い	Narrow	semai	3	7139
Weak	貧しい	Poor	mazushī	4	4590
Weak	鈍い	Dull	nibui	3	2119
Weak	薄い	Thin	usui	3	9364
Weak	臆病	Cowardice*	okubyō	4	27
Weak	女らしい	Feminine	onnarashī	6	106
Weak	脆弱	Fragility*	zeijaku	4	182
Weak	繊細な	Delicate	sensaina	5	–
Weak	病気	Sickness*	byōki	3	16,426
Weak	穏やかな	Quiet	odayakana	5	-
Weak	消極的	Passive	shōkyokuteki	6	885
Weak	小さい	Small	chīsai	4	14,950
Weak	浅はかな	Shallow	asahakana	5	–

Category means word categories used in this study. Positive: positive words; Negative: negative words; Active: active words; Passive: passive words; Strong: strong words; Weak: weak words. We translated English words of previous studies (Staats and Staats 1957; Staats et al. 1959) into Japanese words. There are differences between the part of speech (word class) of some words used in the previous and current studies. * indicates that the word was used as an adjective in Staats and Staats (1957). + indicates that the word was used as a noun in Staats and Staats (1957). Additionally, “sacredness,” “excited,” and “beauty” were used in Survey 1, whereas “sacred,” “excitement,” and “beautiful” were used in Experiment 1 or 2, and Survey 2. Staats and Staats (1957) also used “sacred,” “excited,” and “beauty.” This table shows the words as “sacredness,” “excitement,” and “beautiful.” ** indicates that the word was not used in Staats and Staats (1957) to measure whether the word was strong or weak. The word frequency of fast / quick shows the sum of the word frequency of fast and quick. We counted the word moras. The word frequencies were found in Amano and Kondo (2000). Because sixteen word frequencies could not be found from the database, we showed that “–” means no value in Appendix A.

## Appendix B Means and standard deviations of evaluative responses of words selected for Experiment 1

**Table:**

Positive words (Japanese characters, pronunciation)	*M*	*SD*	Negative words (Japanese characters, pronunciation)	*M*	*SD*
Love (愛, *ai*)	4.68	0.54	Dirty (汚い, *kitanai*)	1.21	0.52
Pretty (可愛い, *kawaii*)	4.63	0.58	Worthless (価値がない, *kachiganai*)	1.35	0.69
Vacation (休暇, *kyūka*)	4.30	0.77	Fear (恐れ, *osore*)	1.85	0.75
Health* (健康, *kenkō*)	4.73	0.63	Insanity* (狂気, *kyōki*)	1.70	0.89
Smart (賢い, *kashikoi*)	4.35	0.63	Bitter (苦い, *nigai*)	2.07	0.69
Happiness* (幸せ, *shiawase*)	4.85	0.40	Stupid (愚かな, *orokana*)	1.39	0.64
Win (勝つ, *katsu*)	4.63	0.55	Disgust* (嫌悪, *ken’o*)	1.35	0.63
Sacredness* (神聖, *shinsei*)	4.33	0.80	Cruelty* (残酷, *zankoku*)	1.42	0.73
Success (成功, *seikō*)	4.82	0.39	Evil (邪悪, *jaaku*)	1.38	0.69
Gift (贈り物, *okurimono*)	4.61	0.54	Ugly (醜い, *minikui*)	1.23	0.52
Valuable (尊い, *tōtoi*)	4.65	0.57	Thief (泥棒, *dorobō*)	1.24	0.46
Beautiful + (美しい, *utsukushī*)	4.68	0.68	Poison (毒, *doku*)	1.47	0.69
Rich (豊か, *yutaka*)	4.74	0.47	Sad (悲しい, *kanashī*)	1.81	0.75
Friend (友達, *tomodachi*)	4.31	0.73	Sickness* (病気, *byōki*)	1.48	0.65

*M*: Mean; *SD*: Standard Deviation; Regarding evaluations of positive and negative words, participants evaluated whether each presented word is positive or negative by using a 5-point semantic differential scale in Survey 1 (1: *negative*; 5: *positive*; Osgood and Suci 1955; Osgood et al. 1957). The means and standard deviations showed evaluative responses of negative and positive words in Survey 1. The selected positive and negative words were used in Experiment 1. These Japanese words were translated from English words in Staats and Staats (1957) by authors who are native speakers of Japanese. There are differences between the part of speech (word class) of some words used in the previous and current studies. * indicates that the word was used as an adjective in Staats and Staats (1957). + indicates that the word was used as a noun in Staats and Staats (1957). Additionally, “sacredness” and “beauty” were used in Survey 1, whereas “sacred” and “beautiful” were used in Experiment 1 and Survey 2. Staats and Staats (1957) also used “sacred” and “beauty.” This table shows the words as “sacredness” and “beautiful.” These words were auditorily presented to each participant in Experiment 1.

## Appendix C Means and standard deviations of evaluative responses of words selected for Experiment 2

**Table:**

Active words (Japanese characters, pronunciation)	*M*	*SD*	Passive words (Japanese characters, pronunciation)	*M*	*SD*
Sharp (鋭い, *surudoi*)	4.12	0.80	Old (お年寄り, *otoshiyori*)	1.96	0.77
Briskness* (活発, *kappatsu*)	4.79	0.54	Calm (穏やか, *odayaka*)	2.34	1.00
Hustle (急かす, *sekasu*)	4.18	0.84	Listless (気だるい, *kedarui*)	1.52	0.74
Hasten + (急ぐ, *isogu*)	4.45	0.70	Resting (休憩, *kyūkei*)	1.89	1.06
Excitement* (興奮, *kōfun*)	4.39	0.66	Lifeless (虚しい, *munashī*)	1.99	0.84
Tension* (緊張, *kinchō*)	3.20	0.94	Laziness* (怠惰, *taida*)	1.29	0.61
Spryness* (元気, *genki*)	4.77	0.47	Slow (遅い, *osoi*)	1.73	0.77
Young (若い, *wakai*)	4.24	0.59	Dull (鈍い, *nibui*)	1.79	0.75
Fast/ quick (速い, *hayai*)	4.67	0.54	Slumber (微睡み, *madoromi*)	1.88	0.83
Hot (熱い, *atsui*)	4.18	0.79	Inertness* (不活性, *fukassei*)	1.54	0.81
Eager (熱心な, *nesshinna*)	4.50	0.63	Peace* (平和, *heiwa*)	2.85	1.05
Ferocity* (猛烈, *mōretsu*)	4.58	0.73	Drowsy (眠い, *nemui*)	1.69	0.87
Agitate (搔き乱す, *kakimidasu*)	3.78	1.13	Cool/relaxed (落ち着いた, *ochitsuita*)	2.40	1.04

*M*: Mean; *SD*: Standard Deviation; Regarding the evaluations of active and passive words, participants evaluated whether each presented word is active or passive by using a 5-point semantic differential scale in Survey 1 (1: *passive*; 5: *active*; Osgood and Suci 1955; Osgood et al. 1957). The means and standard deviations showed evaluative responses of active and passive words in Survey 1. The selected active and passive words were used in Experiment 2. These Japanese words were translated from English words in Staats and Staats (1957) by authors who are native speakers of Japanese. There are differences between the part of speech (word class) of some words used in the previous and current studies. *indicates that the word was used as an adjective in Staats and Staats (1957). + indicates that the word was used as a noun in Staats and Staats (1957). Additionally, “excited” was used in Survey 1, whereas “excitement” was used in Experiment 2 and Survey 2. Staats and Staats (1957) also used “excited.” This table shows the word as “excitement.” These words were auditorily presented to each participant in Experiment 2.

## Appendix D Japanese characters, pronunciations, and English meanings of neutral words used in Experiment 1 and 2

**Table:**

Experiment 1		Experiment 2	
Wordlist A	Wordlist B	Wordlist C	Wordlist D
各地, *kakuchi*, each area	印鑑, *inkan*, name stamp in Japan	印鑑, *inkan*, name stamp in Japan	切符, *kippu*, ticket
切符, *kippu*, ticket	係員, *kakariin*, staff	各地, *kakuchi*, each area	講義, *kōgi*, lecture
景気, *keiki*, economy	講義, *kōgi*, lecture	景気, *keiki*, economy	構造, *kōzō*, construction
国語, *kokugo*, national language	構造, *kōzō*, construction	持参, *jisan*, bringing something	国語, *kokugo*, national language
今度, *kondo*, sometime	持参, *jisan*, bringing something	住所, *jūsho*, home address	今度, *kondo*, sometime
人員, *jin'in*, manpower	住所, *jūsho*, home address	人員, *jin'in*, manpower	状況, *jōkyō*, situation
数字, *sūji*, number	状況, *jōkyō*, situation	選択, *sentaku*, selection	信号, *shingō*, traffic light
選択, *sentaku*, selection	信号, *shingō*, traffic light	台本, *daihon*, script	数字, *sūji*, number
台本, *daihon*, script	同点, *dōten*, tie	男女, *danjo*, men and women	同点, *dōten*, tie
男女, *danjo*, men and women	範囲, *han'i*, range	地域, *chiiki*, region	日時, *nichiji*, date and time
地域, *chiiki*, region	分野, *bunya*, field	道路, *dōro*, road	分野, *bunya*, field
道路, *dōro*, road	面積, *menseki*, area	荷台, *nidai*, load-carrying tray	様子, *yōsu*, state
荷台, *nidai*, load-carrying tray	様子, *yōsu*, state	範囲, *han'i*, range	路線, *rosen*, trainline
日時, *nichiji*, date and time	路線, *rosen*, train line		

These words were selected from a previous study that examined emotional valence and arousal of two-character kanji (Higami et al. 2015). Wordlists of neutral words in Experiment 1 are approximately same with those in Experiment 2, except two words (area and staff). In Experiment 1, participants evaluated whether each presented word is positive or negative by using a 7-point semantic differential scale (1: *negative*; 7: *positive*; Osgood and Suci 1955; Osgood et al. 1957). In Experiment 2, participants evaluated whether each presented word is active or passive by using a 7-point semantic differential scale (1: *passive*; 7: *active*; Osgood and Suci 1955; Osgood et al. 1957). Authors originally translated Japanese words to English meanings for this table. These words were auditorily presented to each participant in Experiments 1 and 2.

## Appendix E Results of Survey 2 to check the positiveness and activeness ratings of positive and negative words used in Experiment 1

**Table:**

Positive words (Japanese characters, pronunciation)	Positiveness		Activeness		Negative words (Japanese characters, pronunciation)	Positiveness		Activeness	
	*M*	*SD*	*M*	*SD*		*M*	*SD*	*M*	*SD*
Love (愛, *ai*)	6.37	0.81	5.00	1.26	Dirty (汚い, *kitanai*)	1.30	0.53	3.23	1.41
Pretty (可愛い, *kawaii*)	6.37	0.89	4.63	0.76	Worthless (価値がない, *kachiganai*)	1.33	0.71	2.17	1.05
Vacation (休暇, *kyūka*)	5.83	0.87	3.83	1.76	Fear (恐れ, *osore*)	1.87	1.17	3.57	1.33
Health* (健康, *kenkō*)	6.23	1.01	5.70	1.15	Insanity* (狂気, *kyōki*)	1.67	0.88	4.77	1.52
Smart (賢い, *kashikoi*)	6.17	0.87	4.93	1.01	Bitter (苦い, *nigai*)	2.60	0.86	3.80	0.92
Happiness* (幸せ, *shiawase*)	6.83	0.38	5.17	1.37	Stupid (愚かな, *orokana*)	1.53	0.82	3.17	1.05
Win (勝つ, *katsu*)	6.37	0.81	6.23	0.97	Disgust* (嫌悪, *ken’o*)	1.30	0.53	3.27	1.36
Sacred (神聖な, *shinseina*)	5.40	0.81	3.37	1.38	Cruelty* (残酷, *zankoku*)	1.23	0.43	3.93	1.28
Success (成功, *seikō*)	6.60	0.67	6.10	0.84	Evil (邪悪, *jaaku*)	1.13	0.35	4.20	1.27
Gift (贈り物, *okurimono*)	5.83	0.91	5.03	1.07	Ugly (醜い, *minikui*)	1.33	0.55	2.80	1.16
Valuable (尊い, *tōtoi*)	6.17	0.79	4.13	1.33	Thief (泥棒, *dorobō*)	1.30	0.53	5.03	1.43
Beautiful + (美しい, *utsukushī*)	6.53	0.63	4.47	1.14	Poison (毒, *doku*)	1.43	0.68	3.40	1.43
Rich (豊か, *yutaka*)	6.30	0.70	5.03	1.19	Sad (悲しい, *kanashī*)	1.57	0.68	3.00	1.26
Friend (友達, *tomodachi*)	5.53	0.82	5.27	0.83	Sickness* (病気, *byōki*)	1.37	0.56	2.50	1.41

*M*: Mean; *SD*: Standard Deviation; First, participants evaluated whether each presented word is positive or negative by using a 7-point semantic differential scale in Survey 2 (1: *negative*; 7: *positive*; Osgood and Suci 1955; Osgood et al. 1957). Second, participants evaluated whether each presented word is active or passive by using a 7-point semantic differential scale in Survey 2 (1: *passive*; 7: *active*; Osgood and Suci 1955; Osgood et al. 1957). The means and standard deviations showed evaluative responses of positive and negative words in Survey 2. The selected positive and negative words were used in Experiment 1. These Japanese words were translated from English words in Staats and Staats (1957) by authors who are native speakers of Japanese. There are differences between the part of speech (word class) of some words used in the previous and current studies. * indicates that the word was used as an adjective in Staats and Staats (1957). + indicates that the word was used as a noun in Staats and Staats (1957). Additionally, “sacredness” and “beauty” were used in Survey 1, whereas “sacred” and “beautiful” were used in Experiment 1 and Survey 2. Staats and Staats (1957) also used “sacred” and “beauty.” This table shows the words “sacred” and “beautiful.” These words were auditorily presented to each participant in Experiment 1.

## Appendix F Results of Survey 2 to check the positiveness and activeness ratings of neutral words used in Experiment 1

**Table:**

Neutral wordlist A: Japanese characters, pronunciations, and English meanings	Positiveness		Activeness		Neutral wordlist B: Japanese characters, pronunciations, and English meanings	Positiveness		Activeness	
	*M*	*SD*	*M*	*SD*		*M*	*SD*	*M*	*SD*
各地, *kakuchi*, each area	4.20	0.48	4.03	1.35	印鑑, *inkan*, name stamp in Japan	3.73	0.64	3.07	1.26
切符, *kippu*, ticket	4.17	0.59	4.30	1.56	係員, *kakariin*, staff	3.90	0.40	4.83	1.15
景気, *keiki*, economy	4.10	0.71	4.83	1.23	講義, *kōgi*, lecture	4.17	0.83	4.50	1.14
国語, *kokugo*, national language	4.20	0.76	3.60	1.13	構造, *kōzō*, construction	4.23	0.43	3.73	1.14
今度, *kondo*, sometime	3.97	0.72	4.03	1.22	持参, *jisan*, bringing something	3.93	0.64	4.90	1.06
人員, *jin'in*, manpower	3.97	0.72	4.47	1.38	住所, *jūsho*, home address	3.90	0.31	3.00	1.36
数字, *sūji*, number	4.07	0.64	3.97	1.52	状況, *jōkyō*, situation	3.87	0.43	4.60	1.33
選択, *sentaku*, selection	4.23	0.68	4.67	1.24	信号, *shingō*, traffic light	3.77	0.82	4.80	1.21
台本, *daihon*, script	3.87	0.57	3.97	1.30	同点, *dōten*, tie	4.17	0.91	4.67	1.21
男女, *danjo*, men and women	4.27	0.74	4.60	1.00	範囲, *han'i*, range	3.93	0.45	3.73	1.17
地域, *chiiki*, region	4.07	0.64	3.93	1.05	分野, *bunya*, field	4.07	0.52	3.83	1.29
道路, *dōro*, road	4.10	0.40	4.40	1.52	面積, *menseki*, area	4.03	0.32	3.17	1.37
荷台, *nidai*, load-carrying tray	3.93	0.58	3.77	1.25	様子, *yōsu*, state	4.07	0.45	4.33	0.99
日時, *nichiji*, date and time	4.03	0.41	4.43	1.25	路線, *rosen*, train line	4.03	0.41	4.60	1.10

These words were selected from a previous study that examined emotional valence and arousal of two-characters kanji (Higami et al. 2015). Wordlists of neutral words in Experiment 1 are approximately same with those in Experiment 2, except two words (area and staff). First, participants evaluated whether each presented word is positive or negative using a 7-point semantic differential scale in Survey 2 (1: *negative*; 7: *positive*; Osgood and Suci 1955; Osgood et al. 1957). Second, participants evaluated whether each presented word is active or passive using a 7-point semantic differential scale in Survey 2 (1: *passive*; 7: *active;* Osgood and Suci 1955; Osgood et al. 1957). The means and standard deviations showed evaluative responses of neutral words in Survey 2. We originally translated Japanese words to English meanings for this table. These words were auditorily presented to each participant in Experiment 1.

## Appendix G Results of Survey 2 to check the positiveness and activeness ratings of active and passive words used in Experiment 2

**Table:**

Active words (Japanese characters, pronunciation)	Positiveness		Activeness		Passive words (Japanese characters, pronunciation)	Positiveness		Activeness	
	*M*	*SD*	*M*	*SD*		*M*	*SD*	*M*	*SD*
Sharp (鋭い, *surudoi*)	4.30	0.84	4.63	1.38	Old (お年寄り, *otoshiyori*)	3.57	0.82	2.57	0.94
Briskness* (活発, *kappatsu*)	6.20	0.85	6.90	0.31	Calm (穏やか, *odayaka*)	5.93	0.83	2.80	1.06
Hustle (急かす, *sekasu*)	2.43	0.63	6.10	1.24	Listless (気だるい, *kedarui*)	2.40	1.04	1.80	0.76
Hasten + (急ぐ, *isogu*)	3.43	0.68	6.30	1.09	Resting (休憩, *kyūkei*)	5.33	1.06	2.10	1.32
Excitement* (興奮, *kōfun*)	5.03	0.85	6.23	0.97	Lifeless (虚しい, *munashī*)	1.73	0.74	2.00	0.98
Tension* (緊張, *kinchō*)	2.87	0.82	3.93	1.20	Laziness* (怠惰, *taida*)	1.67	0.76	1.43	0.68
Spryness* (元気, *genki*)	6.50	0.63	6.73	0.52	Slow (遅い, *osoi*)	2.37	0.85	2.10	0.84
Young (若い, *wakai*)	5.60	0.89	6.13	1.01	Dull (鈍い, *nibui*)	2.37	0.85	2.07	1.05
Fast/ quick (速い, *hayai*)	5.33	0.84	6.47	0.78	Slumber (微睡み, *madoromi*)	4.40	1.16	2.70	1.21
Hot (熱い, *atsui*)	4.60	0.93	5.57	1.01	Inertness* (不活性, *fukassei*)	2.40	1.13	1.53	0.78
Eager (熱心な, *nesshinna*)	5.73	0.87	6.27	0.87	Peace* (平和, *heiwa*)	6.43	0.90	4.00	1.20
Ferocity* (猛烈, *mōretsu*)	4.23	0.94	6.43	0.82	Drowsy (眠い, *nemui*)	3.40	0.86	1.77	0.86
Agitate (搔き乱す, *kakimidasu*)	2.07	1.11	5.77	1.41	Cool/relaxed (落ち着いた, *ochitsuita*)	5.60	0.86	2.50	1.22

*M*: Mean; *SD*: Standard Deviation; First, participants evaluated whether each presented word is positive or negative by using a 7-point semantic differential scale in Survey 2 (1: *negative*; 7: *positive*; Osgood and Suci 1955; Osgood et al. 1957). Second, participants evaluated whether each presented word is active or passive by using a 7-point semantic differential scale in Survey 2 (1: *passive*; 7: *active*; Osgood and Suci 1955; Osgood et al. 1957). The means and standard deviations showed evaluative responses of active and passive words in Survey 2. The selected active and passive words were used in Experiment 2. These Japanese words were translated from English words in Staats and Staats (1957) by authors who are Japanese native speakers. The part of speech (word class) of some words used in the previous and current studies differs. * indicates that the word was used as an adjective in Staats and Staats (1957). + indicates that the word was used as a noun in Staats and Staats (1957). Additionally, “excited” was used in Survey 1, whereas “excitement” was used in Experiment 2 and Survey 2. Staats and Staats (1957) also used “excited.” This table presents the word as “excitement.” These words were auditorily presented to each participant in Experiment 2.

## Appendix H Results of Survey 2 to check positiveness and activeness ratings of neutral words used in Experiment 2

**Table:**

Neutral wordlist A: Japanese characters, pronunciations, and English meanings	Positiveness		Activeness		Neutral wordlist B: Japanese characters, pronunciations, and English meanings	Positiveness		Activeness	
	*M*	*SD*	*M*	*SD*		*M*	*SD*	*M*	*SD*
印鑑, *inkan*, name stamp in Japan	3.73	0.64	3.07	1.26	切符, *kippu*, ticket	4.17	0.59	4.30	1.56
各地, *kakuchi*, each area	4.20	0.48	4.03	1.35	講義, *kōgi*, lecture	4.17	0.83	4.50	1.14
景気, *keiki*, economy	4.10	0.71	4.83	1.23	構造, *kōzō*, construction	4.23	0.43	3.73	1.14
持参, *jisan*, bringing something	3.93	0.64	4.90	1.06	国語, *kokugo*, national language	4.20	0.76	3.60	1.13
住所, *jūsho*, home address	3.90	0.31	3.00	1.36	今度, *kondo*, sometime	3.97	0.72	4.03	1.22
人員, *jin'in*, manpower	3.97	0.72	4.47	1.38	状況, *jōkyō*, situation	3.87	0.43	4.60	1.33
選択, *sentaku*, selection	4.23	0.68	4.67	1.24	信号, *shingō*, traffic light	3.77	0.82	4.80	1.21
台本, *daihon*, script	3.87	0.57	3.97	1.30	数字, *sūji*, number	4.07	0.64	3.97	1.52
男女, *danjo*, men and women	4.27	0.74	4.60	1.00	同点, *dōten*, tie	4.17	0.91	4.67	1.21
地域, *chiiki*, region	4.07	0.64	3.93	1.05	日時, *nichiji*, date and time	4.03	0.41	4.43	1.25
道路, *dōro*, road	4.10	0.40	4.40	1.52	分野, *bunya*, field	4.07	0.52	3.83	1.29
荷台, *nidai*, load-carrying tray	3.93	0.58	3.77	1.25	様子, *yōsu*, state	4.07	0.45	4.33	0.99
範囲, *han'i*, range	3.93	0.45	3.73	1.17	路線, *rosen*, trainline	4.03	0.41	4.60	1.10

These words were selected from a previous study that examined emotional valence and arousal of two-characters kanji (Higami et al. 2015). Wordlists of neutral words in Experiment 2 are approximately same with those in Experiment 1, except two words (area and staff). First, participants evaluated whether each presented word is positive or negative using a 7-point semantic differential scale in Survey 2 (1: *negative*; 7: *positive*; Osgood and Suci 1955; Osgood et al. 1957). Second, participants evaluated whether each presented word is active or passive using a 7-point semantic differential scale in Survey 2 (1: *passive*; 7: *active*; Osgood and Suci 1955; Osgood et al. 1957). The means and standard deviations showed evaluative responses of neutral words in Survey 2. We originally translated Japanese words to English meanings for this table. These words were auditorily presented to each participant in Experiment 2.
