# Correction to: Japanese written pseudowords can be conditioned to Japanese spoken words with positive, negative, and active emotions

**DOI:** 10.1007/s10339-024-01174-4

**Published:** 2024-02-07

**Authors:** Misa Ando, Toshimune Kambara

**Affiliations:** Department of Psychology, Graduate School of Humanities and Social Sciences, Hiroshima University, Higashihiroshima, Japan

**Correction to: Cognitive Processing (2023) 24:387–413** 10.1007/s10339-023-01138-0

The article “Japanese written pseudowords can be conditioned to Japanese spoken words with positive, negative, and active emotions,” written by Misa Ando and Toshimune Kambara, was originally published Online First without Open Access. After publication in volume 24, issue 3, page 387–413, the author decided to opt for Open Choice and to make the article an Open-Access publication. Therefore, the copyright of the article has been changed to © The Author(s) 2024, and the article is forthwith distributed under the terms of the Creative Commons Attribution 4.0 International License, which permits use, sharing, adaptation, distribution, and reproduction in any medium or format, as long as you give appropriate credit to the original author(s) and the source, provide a link to the Creative Commons licence, and indicate if changes were made. The images or other third-party material in this article are included in the article’s Creative Commons licence, unless indicated otherwise in a credit line to the material. If material is not included in the article’s Creative Commons licence and your intended use is not permitted by statutory regulation or exceeds the permitted use, you will need to obtain permission directly from the copyright holder. To view a copy of this licence, visit http://creativecommons.org/licenses/by/4.0.

Original article has been corrected.

